# Deep Sequencing and Ecological Characterization of Gut Microbial Communities of Diverse Bumble Bee Species

**DOI:** 10.1371/journal.pone.0118566

**Published:** 2015-03-13

**Authors:** Haw Chuan Lim, Chia-Ching Chu, Manfredo J. Seufferheld, Sydney A. Cameron

**Affiliations:** 1 Department of Entomology, University of Illinois, Urbana, IL, 61801, United States of America; 2 Department of Crop Sciences, University of Illinois, Urbana, IL, 61801, United States of America; Universidade de São Paulo, Faculdade de Filosofia Ciências e Letras de Ribeirão Preto, BRAZIL

## Abstract

Gut bacterial communities of bumble bees are correlated with defense against pathogens. Further understanding this host-microbe association is vitally important as bumble bees are currently experiencing global population declines, potentially due in part to emergent diseases. In this study, we used pyrosequencing and community fingerprinting (ARISA) to characterize the gut microbial communities of nine bumble species from across the *Bombus* phylogeny. Overall, we delimited 74 bacterial taxa (operational taxonomic units or OTUs) belonging to Betaproteobacteria, Gammaproteobacteria, Bacilli, Actinobacteria, Flavobacteria and Alphaproteobacteria. Each bacterial community was taxonomically simple, containing an average of 1.9 common (relative abundance per sample > 5%) bacterial OTUs. The most abundant and prevalent (occurring in 92% of the samples) bacterial OTU, based on 16S rRNA sequences, closely matched that of the previously described Betaproteobacteria species *Snodgrassella alvi*. Bacteria that were first described in bee-related external environments dominated a number of gut bacterial communities, suggesting that they are not strictly dependent on the internal gut environment. The ARISA data showed a correlation between bacterial community structures and the geographic locations where the bees were sampled, suggesting that at least a subset of the bacterial species may be transmitted environmentally. Using light and fluorescent microscopy, we demonstrated that the gut bacteria form a biofilm on the internal epithelial surface of the ileum, corroborating results obtained from *Apis mellifera*.

## Introduction

Recent studies have shown that bacterial symbionts of animals play significant roles in shaping the physiology, ecology and evolution of their hosts [1,2]. In insects, these symbionts allow specialization on particular diets, such as termites on wood and aphids on phloem, even to the extent that some host and bacterial genomes have become complementary and compensatory with respect to genes that underlie biosynthesis pathways [3,4]. Moreover, resident gut bacteria may protect hosts against invasion by microbial pathogens through production of antimicrobial compounds, stimulation of host-immune systems or competition for space and nutrients [5–7]. Such colonization resistance conferred by resident microbiota has been documented in a wide variety of insects, including flies [8], caterpillars [9], termites [10], and locusts [11] and bumble bees [12].

Recent studies of gut microbiota in bees belonging to the corbiculate clade (honey bees [Apini], bumble bees [Bombini], stingless bees [Meliponini] and orchid bees [Euglossini]) have advanced understanding of the taxonomic composition and structure of microbial communities [13–15], elucidated important health effects and roles in defense against host enemies [12,16–19] and revealed ecological factors, such as host species, and environmental chemical substances that affect bacterial communities [20,21]. Understanding the contributions of intestinal microbiota to host fitness of bees is important and timely as many species of these globally important pollinators are currently under threat from disease [22–25], agrochemicals [26,27], habitat degradation [28,29] or a combination of these factors [30,31]. To date, most studies of bumble bees (*Bombus*), which are much more speciose than honey bees (*Apis*), have focused on specific bacterial lineages [32] of only a few host species [15], often relying on 16S rRNA sequencing of a small number of molecular clones, limiting the scope of our knowledge of the microbial diversity and structure of their gut microbiota [33]. To further our understanding of the composition and structure of bumble bee gut bacterial communities, we conducted pyrosequencing and community fingerprinting of gut bacterial microbiota of nine bumble bee species from across the known *Bombus* phylogeny—to date, the gut microbiota of most of our study species have not been characterized at all. We applied naïve Bayesian classification and phylogenetic analysis to the pyrosequences. To examine the effects of bumble bee species and sampling locality on bacterial community profile, we conducted community fingerprinting on a subset of taxa, using larger sampling of each species from more localities. Finally, we used light and fluorescent microscopy to visualize the distribution of bacteria in the lumen of the gut of *Bombus impatiens* to compare with the pattern reported for honey bees [34].

## Materials and Methods

### Sampling of bumble bees

To conduct gut bacterial 16S rRNA amplicon sequencing and Automated Ribosomal Intergenic Spacer Analysis (ARISA), we assembled a total of 57 individuals of nine species (*B*. *atratus*, *B*. *bohemicus*, *B*. *convexus*, *B*. *fraternus*, *B*. *impatiens*, *B*. *pensylvanicus*, *B*. *terrestris*, *B*. *terricola* and *B*. *ussurensis*) from seven subgenera. These include species that are known to be undergoing widespread or regional population declines in the US (*B*. [*Thoracobombus*] *pensylvanicus*, *B*. [*Bombus*] *terricola* and *B*. [*Cullumanobombus*] *fraternus*) [35,36], a US species with healthy populations (*B*. [*Pyrobombus*] *impatiens*) [35], a European species used for commercial pollination (*B*. [*Bombus*] *terrestris*), a socially parasitic species (*B*. [*Psithyrus*] *bohemicus*) and two early diverging species (*B*. [*Mendacibombus*] *convexus* and *B*. [*Diversobombus*] *ussurensis*) [37] (Table 1). All individuals were workers collected in the field with aerial nets. For 16S rRNA amplicon sequencing, we examined all nine species to understand the taxonomic diversity of bacteria found in taxa selected from different clades across the *Bombus* phylogeny. For this portion of the study, we sequenced 1–2 pools of whole-gut DNA for each species. Each pool of DNA was the result of equimolar mixing of DNA from two to three conspecific bees that were collected from the same locality at the same time (Table 1). Hereafter, each such pool will be referred to as a pyrosequencing sample. In total, 29 individuals from 13 distinct locations were used for sequencing, resulting in 13 pyrosequencing samples. The sequencing IDs for the samples are capitalized and enclosed within quotes in the following sections. To complement the amplicon sequencing, we used ARISA—a bacterial community fingerprinting technique relying on length variation in the rRNA internal transcribed spacer—to examine the effects of host species and geography on dissimilarity in bacterial communities. We applied ARISA to gut bacterial communities from four North American species (*B*. *fraternus*, *B*. *impatiens*, *B*. *pensylvanicus*, *B*. *terricola*) and one from Europe (*B*. *terrestris*), samples of which were taken from 2–3 different geographic locations. A total of 44 individuals were used for ARISA. Individuals were treated separately in this portion of the study (i.e., DNA from multiple individuals was not pooled) and each bee was considered an independent sample (Table 1).

**Table 1:** Bumble bees used in amplicon sequencing and ARISA, and their geographic origins.

ARISA ID	Pyrosequencing sample ID	*Bombus* species	State/Province	Country	Latitude	Longitude	Field ID (if available)
	*ATRATUS*	*B*. *atratus*	Boyaca	Columbia	5.64	-72.9	
	*ATRATUS*	*B*. *atratus*	Boyaca	Columbia	5.64	-72.9	
	*BOHEMICUS*	*B*. *bohemicus*	Uppland	Sweden	60.48	17.62	
	*BOHEMICUS*	*B*. *bohemicus*	Uppland	Sweden	60.48	17.62	
	*BOHEMICUS*	*B*. *bohemicus*	Uppland	Sweden	60.48	17.62	
	*CONVEXUS*	*B*. *convexus*	Sichuan	China	32.79	102.54	
	*CONVEXUS*	*B*. *convexus*	Sichuan	China	32.79	102.54	
	*CONVEXUS*	*B*. *convexus*	Sichuan	China	32.79	102.54	
B.FR7	*FRATERNUS_FL*	*B*. *fraternus*	Florida	USA	30.13	-83.35	FL11.0011
B.FR9	*FRATERNUS_FL*	*B*. *fraternus*	Florida	USA	30.13	-83.35	FL11.0009
B.FR10		*B*. *fraternus*	Florida	USA	30.13	-83.35	FL11.0010
B.FR2		*B*. *fraternus*	Georgia	USA	31.35	-82.02	GA11.0031
B.FR3		*B*. *fraternus*	Georgia	USA	31.35	-82.02	GA11.0028
B.FR4		*B*. *fraternus*	Georgia	USA	31.35	-82.02	GA11.0032
B.FR6		*B*. *fraternus*	North Carolina	USA	34.67	-77.03	NC11.0032
B.FR8		*B*. *fraternus*	North Carolina	USA	34.67	-77.03	NC11.0036
B.IM10	*IMPATIENS_GA*	*B*. *impatiens*	Georgia	USA	31.85	-82.36	GA11.0015
B.IM12	*IMPATIENS_GA*	*B*. *impatiens*	Georgia	USA	31.85	-82.36	GA11.0014
B.IM3	*IMPATIENS_MI*	*B*. *impatiens*	Michigan	USA	44.98	-84.28	MI11.0028
	*IMPATIENS_MI*	*B*. *impatiens*	Michigan	USA	44.98	-84.28	MI11.0031
B.IM1		*B*. *impatiens*	Michigan	USA	44.98	-84.28	MI11.0030
B.IM2		*B*. *impatiens*	Michigan	USA	44.98	-84.28	MI11.0029
B.IM5		*B*. *impatiens*	Michigan	USA	44.98	-84.28	MI11.0032
B.IM6		*B*. *impatiens*	West Virginia	USA	37.77	-80.23	WV11.0007
B.IM8		*B*. *impatiens*	West Virginia	USA	37.77	-80.23	WV11.0004
B.IM9		*B*. *impatiens*	West Virginia	USA	37.77	-80.23	WV11.0006
B.PE4	*PENSYLVANICUS_FL*	*B*. *pensylvanicus*	Florida	USA	29.89	-82.67	FL11.0001
	*PENSYLVANICUS_FL*	*B*. *pensylvanicus*	Florida	USA	29.89	-82.67	FL11.0003
B.PE1	*PENSYLVANICUS_NC*	*B*. *pensylvanicus*	North Carolina	USA	34.48	-78.63	NC11.0042
B.PE5	*PENSYLVANICUS_NC*	*B*. *pensylvanicus*	North Carolina	USA	34.48	-78.63	NC11.0040
B.PE2		*B*. *pensylvanicus*	Florida	USA	29.89	-82.67	FL11.0002
B.PE3		*B*. *pensylvanicus*	South Carolina	USA	34.23	-79.15	SC11.0005
B.PE6		*B*. *pensylvanicus*	South Carolina	USA	34.23	-79.15	SC11.0004
B.PE9		*B*. *pensylvanicus*	South Carolina	USA	34.23	-79.15	SC11.0003
B.TRS.7	*TERRESTRIS_ARG*	*B*. *terrestris*	Río Negro	Argentina	-43.06	-71.53	BARI2012.003
B.TRS.8	*TERRESTRIS_ARG*	*B*. *terrestris*	Río Negro	Argentina	-43.06	-71.53	BARI2012.004
B.TRS.1	*TERRESTRIS_FR*	*B*. *terrestris*	Pyrenees-Orientalis	France	42.55	2.97	Fr10.350
B.TRS.2	*TERRESTRIS_FR*	*B*. *terrestris*	Pyrenees-Orientalis	France	42.55	2.97	Fr10.351
B.TRS.3		*B*. *terrestris*	Pyrenees-Orientalis	France	42.55	2.97	Fr10.347
B.TRS.4		*B*. *terrestris*	Pyrenees-Orientalis	France	42.55	2.97	Fr10.352
B.TRS.5		*B*. *terrestris*	Río Negro	Argentina	-43.06	-71.53	BARI2012.001
B.TRS.6		*B*. *terrestris*	Río Negro	Argentina	-43.06	-71.53	BARI2012.002
B.TE10	*TERRICOLA_MI*	*B*. *terricola*	Michigan	USA	46.16	-88.77	MI11.0014
B.TE14	*TERRICOLA_MI*	*B*. *terricola*	Michigan	USA	46.16	-88.77	MI11.0001
B.TE12	*TERRICOLA_WI*	*B*. *terricola*	Wisconsin	USA	45.73	-89.54	WI11.0002
B.TE2	*TERRICOLA_WI*	*B*. *terricola*	Wisconsin	USA	45.73	-89.54	WI11.0009
B.TE13		*B*. *terricola*	Michigan	USA	46.16	-88.77	MI11.0002
B.TE5		*B*. *terricola*	Michigan	USA	46.16	-88.77	MI11.0018
B.TE3		*B*. *terricola*	Minnesota	USA	47.89	-92.77	MN11.0043
B.TE4		*B*. *terricola*	Minnesota	USA	47.89	-92.77	MN11.0027
B.TE6		*B*. *terricola*	Minnesota	USA	47.89	-92.77	MN11.0026
B.TE7		*B*. *terricola*	Minnesota	USA	47.89	-92.77	MN11.0038
B.TE9		*B*. *terricola*	Minnesota	USA	47.89	-92.77	MN11.0040
B.TE1		*B*. *terricola*	Wisconsin	USA	45.73	-89.54	WI11.0008
	*USSURENSIS*	*B*. *ussurensis*	North Chungcheong	South Korea	38.1	127.07	
	*USSURENSIS*	*B*. *ussurensis*	North Chungcheong	South Korea	38.1	127.07	
	*USSURENSIS*	*B*. *ussurensis*	North Chungcheong	South Korea	38.1	127.07	

For amplicon sequencing, bumble bees whose DNAs were pooled prior to Roche 454 library preparation were given the same pyrosequencing sample ID.

### Ethics statement

Our collections included no endangered invertebrate species. No national permits were required to collect the bumble bee samples from public lands in the United States, Europe, Argentina, and other locations. For the samples collected from China, permits were obtained via Dr. Xue-xin Chen, Professor of Entomology, Institute of Insect Science, Zhejiang University, Zijingang Campus, Hangzhou 310058, China.

### Gut and DNA extraction

Prior to abdomen excision, each bee was kept either in 95% (vol/vol) ethanol at 4°C or at −80°C. To remove the entire gut from each bee, we surface-sterilized the bee in 70% alcohol for 30 s. This was followed by a 5-min washing in 0.1% of Triton-X-100 and two rinses in distilled water. Gut tissue was then excised in Ringer’s solution under a stereomicroscope. Total DNA from the excised gut was extracted using the FastDNA SPIN kit for soil (MP Biomedicals).

### Pyrosequencing library preparation

Based on Wang and Qian [38], we designed and optimized primers that target the V4-V6 variable regions of bacterial 16S rRNA. These primers (E515f: GTGCCAGCAGCCGCGGTA and E1063r: CTCACGRCACGAGCTGACG) span approximately 567 bp of the rRNA and produce amplicon sizes that are suitable for Roche 454 GS FLX+ sequencing. Further, these variable rRNA regions have been shown to be useful for taxonomic assignments and phylogenetic placement [39,40]. To produce sequencing libraries, we conducted two rounds of PCR using pooled DNA as templates (for details, see supporting information). For the first round of PCR, we used basic primers (as listed above) with universal tails attached to the 5’ ends (for E515f, GTTGTAAAACGACGGCCAGT, and for E1063r, CACAGGAAACAGCTATGACC). Upon completion of the first round of PCR, we took 5–10 ng of the product and conducted a second PCR to attach Roche 454 Lib-L sequencing primers and barcodes for identification. After gel extraction (eventual amplicon size after round-two PCR ~677 bp) and quality assessment, the libraries were submitted to the University of Illinois W. M. Keck Center for Comparative and Functional Genomics for 1/4^th^ PTP sequencing on a Roche 454 GS FLX+ machine.

### Pyrosequencing data processing

After preliminary signal processing using Roche software v. 2.6, we used the mothur v 1.2 [41] suite of modules to conduct demultiplexing, quality filtering, and data processing and analysis (see supporting information for details). To align and subsequently classify the 16S rRNA sequences generated, we used a custom, taxonomically annotated 16S rRNA training set composed of: 1) a SILVA reference alignment (14,956 sequences, downloaded from http://www.mothur.org in September 2012) and 2) an alignment of 16S rRNA sequences (*n* = 276) derived from honey bee (*Apis mellifera*) [42]. After chimera screening and weeding, we carried out taxonomic classification of the remaining sequences using the Ribosomal Database Project’s (RDP) naïve Bayesian classifier [40]. To assess the confidence of each taxonomic designation, we conducted bootstrap confidence estimation (1,000 iterations) following Wang et al. [40]. Sequences with taxonomic designations matching Cyanobacteria or chloroplast (likely contaminant sequences derived from pollens) and those represented by only one read (singletons) were removed. The remaining sequences were used to conduct average neighbor clustering [41] to assign sequences to clusters (distance threshold = 3%). From each OTU, we randomly selected one representative read and used it to conduct taxonomic classification to the family level using RDP’s naïve Bayesian Classifier described above. In our custom training set, we followed a previous study [42], regarding bacterial phylotypes or clades named in honey bees (e.g., alpha-2.1), as representing family groups. We used the customized training set to provide a general picture of the classification of delimited OTUs to the family level. For OTUs with the highest relative abundance in each pyrosequencing sample, we took sequences of dominant OTUs and conducted BLASTn searches against the GenBank nr database.

### 16S rRNA rarefaction, phylogenetic and community analyses

To assess the completeness of bacterial OTU sampling, we carried out intra- and inter-sample rarefaction analysis (details in supporting information). To estimate a phylogeny of the 74 bacterial OTUs, we aligned the representative sequence for each OTU and aligned it against 276 near full-length 16S rRNA sequences from a previous study [42] using a template-based alignment method implemented in CRWAlign [43]. Two sequences (CP000459 and CP001562) from their original set could not be aligned using this method. Next, we used RNAalifold [44] to predict consensus secondary structure based on the aligned sequences (RIBOSUM scoring, other settings default) and incorporated this information into MrBayes 3.2 [45] for phylogeny estimation. Specifically, we identified RNA stem-pairs as those receiving covariance scores greater than or equal to 90, and coded them as such under MrBayes’ doublet model (nucmodel = doublet); the remaining nucleotide bases were placed in another data partition with the following evolutionary parameters: nucmodel = 4by4, nst = 6 and rates = invgamma. We ran MrBayes for five million generations (sufficient to reach stationarity, split frequencies < 0.05), two parallel runs, three heated chains and one cold chain per run, sampling the chains every 1,000 generations, and calculated the 50% majority-rule consensus tree. Liu et al. [46] showed that short reads, when added to a phylogeny based on full length sequences, can be placed accurately within the phylogeny.

We used the Unique Fraction (UniFrac) [47] metric to assess inter-sample microbial differences. This method employs information contained within the microbial phylogeny (i.e., the Bayesian consensus tree) to determine the fraction of evolution (branch length) that is unique to each microbial community under investigation. We performed the “all environments together” and the “each environment individually” UniFrac tests using the program’s web server [48]. The “all environments together” test determined if sequences differed significantly across all 13 pyrosequencing samples while the “each environment individually” test determined if each community was significantly different from the remaining ones. In each case, statistical significance was established by comparing the observed UniFrac distance against distances obtained when community membership of sequences was randomized (number of permutations = 100). We further calculated normalized weighted inter-sample UniFrac distances and used these distances to conduct non-metric multidimensional scaling (NMDS) and hierarchical clustering in mothur [41]. To assess robustness of the cluster analysis, we conducted jackknife resampling, keeping 972 sequences (= 75% of the total number of sequences in the sample with the fewest reads [“*BOHEMICUS*”]) for each repetition (100 total repetitions).

### ARISA protocol and data analysis

We conducted ARISA following Fisher and Triplett [49] with slight modifications. We carried out PCR amplifications on 30 ng of DNA using primer pair ITSF and ITSReub from a previous study [50]. Primer ITSF was 5’-labeled with the phosphoramidite dye 6-FAM. PCR conditions were set at 94°C-2 min, followed by 30 cycles of 94°C-45 s, 55°C-45 s, 72°C-1 min, and 72°C-10 min. Amplified samples were subjected to capillary electrophoresis-based fragment analysis with an ABI 3730xl genetic analyzer (Applied Biosystems Inc.). We used the Genemapper software (Applied Biosystems Inc.) to output fluorescence intensity data of all fragments that fell between 0.1 and 1 kb. We conducted noise filtering (peak height, standard deviation multiplier = 2) and fragment binning (clustering threshold = 1.2 bp) with the T-REX software [51]. Hellinger transformation [52] was applied to the dataset and transformed data were used to calculate among-sample Bray-Curtis dissimilarity measures using the “vegan” package in R [53]. Multivariate analyses of the dissimilarity measures were conducted using PRIMER6 and PERMANOVA+ (PRIMER-E Ltd.) [54]. Non-metric multidimensional scaling was used to project the relationships among samples into lower dimensional space. To study factors influencing gut microbiotas of bumble bees, we conducted permutational multivariate analysis of variance (PERMANOVA) using an unbalanced nested model that included host species (fixed effect), and sampling location (random effect nested in species) as factors [54]. The PERMANOVA test was conducted using type III sums of squares; permutations (9,999 iterations) of residuals were conducted under a reduced model.

### Light and fluorescent microscopy of bumble bee gut tissues

To visualize bacteria located in portions of the ileum of bumble bees using light and fluorescent microscopy, we dissected randomly selected workers from a commercial *B*. *impatiens* colony obtained from Koppert Biological Systems Inc. The relatively abundant *B*. *impatiens* was chosen as a representative species of the genus. A previous study of honey bees showed that the ileum (together with the rectum) was a key part of the intestinal tract that harbored bacteria (bacteria were largely absent from the crop and the ventriculus/midgut) [34]. Details regarding gut sample preparation are described in the supporting information. Both light and fluorescent images were taken with a Nikon E600 microscope with universal objective lenses.

### DNA sequence accession numbers

Representative sequences of the OTUs are available in GenBank under the following accession numbers: KP412082−KP412155.

## Results

### Bacterial phylogeny and taxonomic classification

After data quality control and filtering, we obtained a total of 106,196 pyrosequencing reads of bacterial 16S rRNA, with an average read length of 445.0 bp. Seventy four bacterial OTUs were delimited, and the number of reads included under each OTU ranged from two to 57,931 (S1 Table). Representative sequences of OTUs received high-confidence taxonomic assignments from the naïve Bayesian classifier. The average bootstrap values at the levels of Class, Order and Family were 92.4%, 88.6% and 83.5%, respectively (S1 Table). These values do not include samples that were not classified. The proportion of representative sequences lacking classification was highest at the level of Family (35.1%), followed by Order (2.7%) and Class (1.4%), reflecting our generally poor understanding of bacterial taxonomy at lower taxonomic levels (S1 Table and Table 2). One representative sequence was assigned with very low confidence to an unclassified bacterium clone (ML635J-21) that originated from a meromictic soda lake [55].

**Table 2:** Results of taxonomic classification (Class to Family) conducted on representative sequences of 74 bacterial OTUs.

Class	Order	Family	Number of OTUs with this Classification	Relative abundance (%) averaged across 13 pyrosequencing samples
Actinobacteria	Bifidobacteriales	unclassified	11	2.746
Alphaproteobacteria	Rhodospirillales	Acetobacteraceae	1	3.800
		**alpha-2.2**	1	0.002
Bacilli	Lactobacillales	**firm-5**	1	0.123
		Lactobacillaceae	2	0.007
		Leuconostocaceae	3	3.933
		unclassified	13	14.424
	unclassified	unclassified	1	0.003
Betaproteobacteria	Burkholderiales	Burkholderiaceae	1	0.064
	Neisseriales	**beta**	19	52.594
Flavobacteria	Flavobacteriales	Flavobacteriaceae	3	8.805
Gammaproteobacteria	Enterobacteriales	Enterobacteriaceae	9	2.315
		**gamma-1**	5	10.314
	Oceanospirillales	Oceanospirillaceae	3	0.869
ML635J-21	unclassified	unclassified	1	0.002

Families listed in bold correspond to phylotypes typically found in western honey bees (*Apis mellifera*).

**Fig 1:**
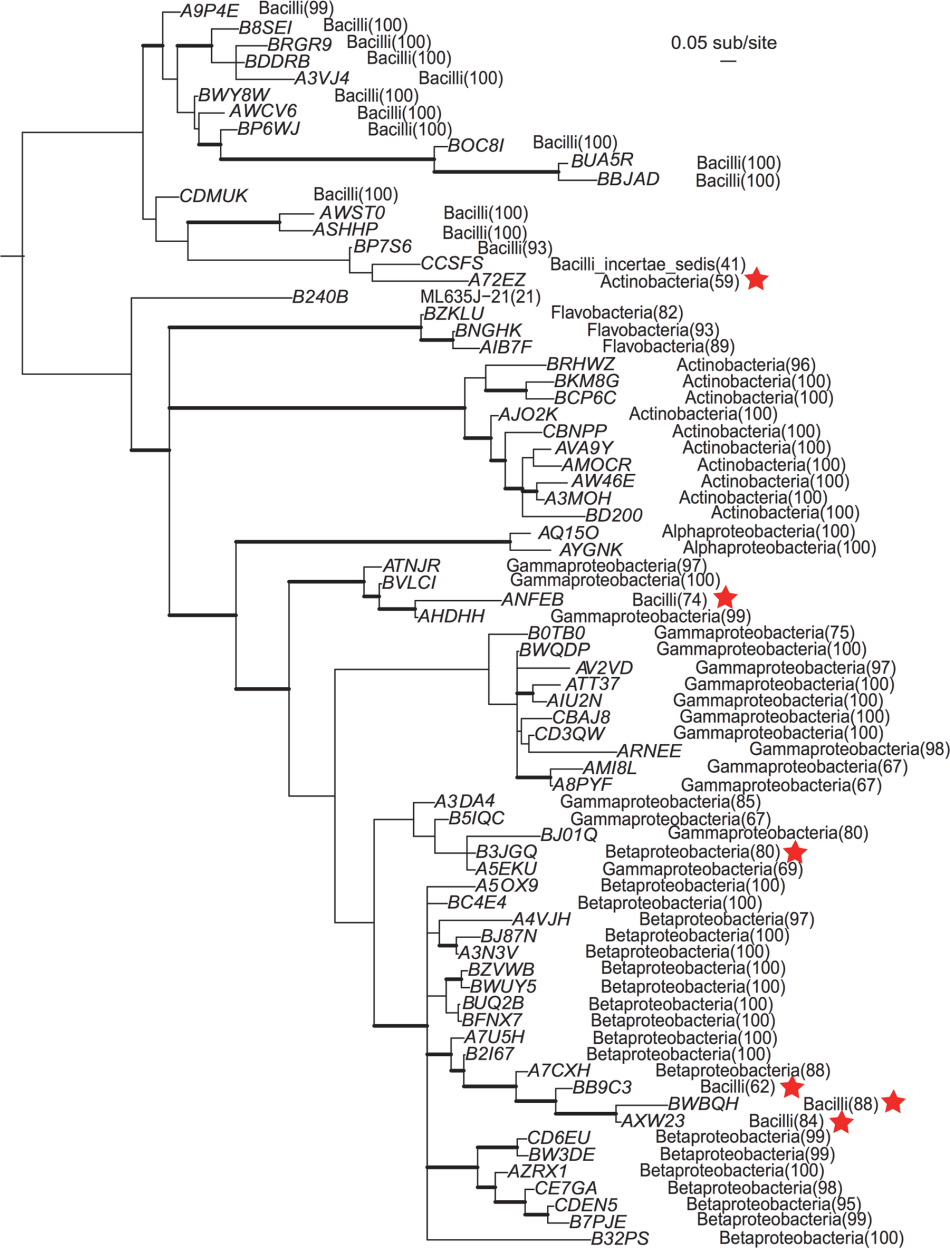
Consensus Bayesian inference tree showing phylogenetic relationships of representative sequences of 74 bacterial OTUs, and their classifications at the level of Class (classification bootstrap confidence in parenthesis). The IDs of the OTUs are abbreviated by only keeping their unique, last five characters (e.g., HUSOE0I01*A9P4E* to *A9P4E*). The tree was inferred by combining these sequences with 274 16S rRNA sequences from [42], followed by dropping the latter sequences from the tree using the drop.tip function found in the R package “ape”. Thickened branches indicate posterior probabilities of 90% or higher; stars signify sequences that have classification-phylogeny incompatibility. See S1 Fig. for classifications of the OTUs at various taxonomic levels. See S2 Fig. for the full trees that include sequences from [42].

Concordance between the taxonomy assigned to sequences and their placement in the bacterial phylogeny was strong, as sequences possessing the same taxonomic classifications generally clustered together in the tree (Fig. 1, S1 Fig. and S2 Fig.). This was especially true for the full-length sequences from a previous work [42], where there were no classification-phylogeny mismatches at any taxonomic level, except for one case (at the Family level) in which a sequence assigned to Anaplasmataceae was phylogenetically nested within Rickettsiaceae, a sister Family within the same Order, Rickettsiales (S2 Fig.). Sequences assigned to Family-level bacterial phylotypes commonly found in honey bees were found in expected positions in the phylogeny. For example, sequences assigned to alpha-2.2 were found within Rhodospirillales, and those assigned to beta were found within Neisseriales (S1 Fig.). Of the 74 bacterial OTUs we delimited, representative sequences from only six (6.1%) showed classification-phylogeny mismatches, indicating that the short sequences made it more difficult to conduct classification and/or phylogeny placement (Fig. 1). These six sequences received relatively low bootstrap confidence scores from the naïve Bayesian classifier (at the Class level, average = 74.5%). Sequences belonging to OTUs with mismatches constitute a small portion of the total pool (0.24%) and thus have negligible effects on downstream analysis (S1 Table).

### Sampling adequacy and community diversity

**Fig 2:**
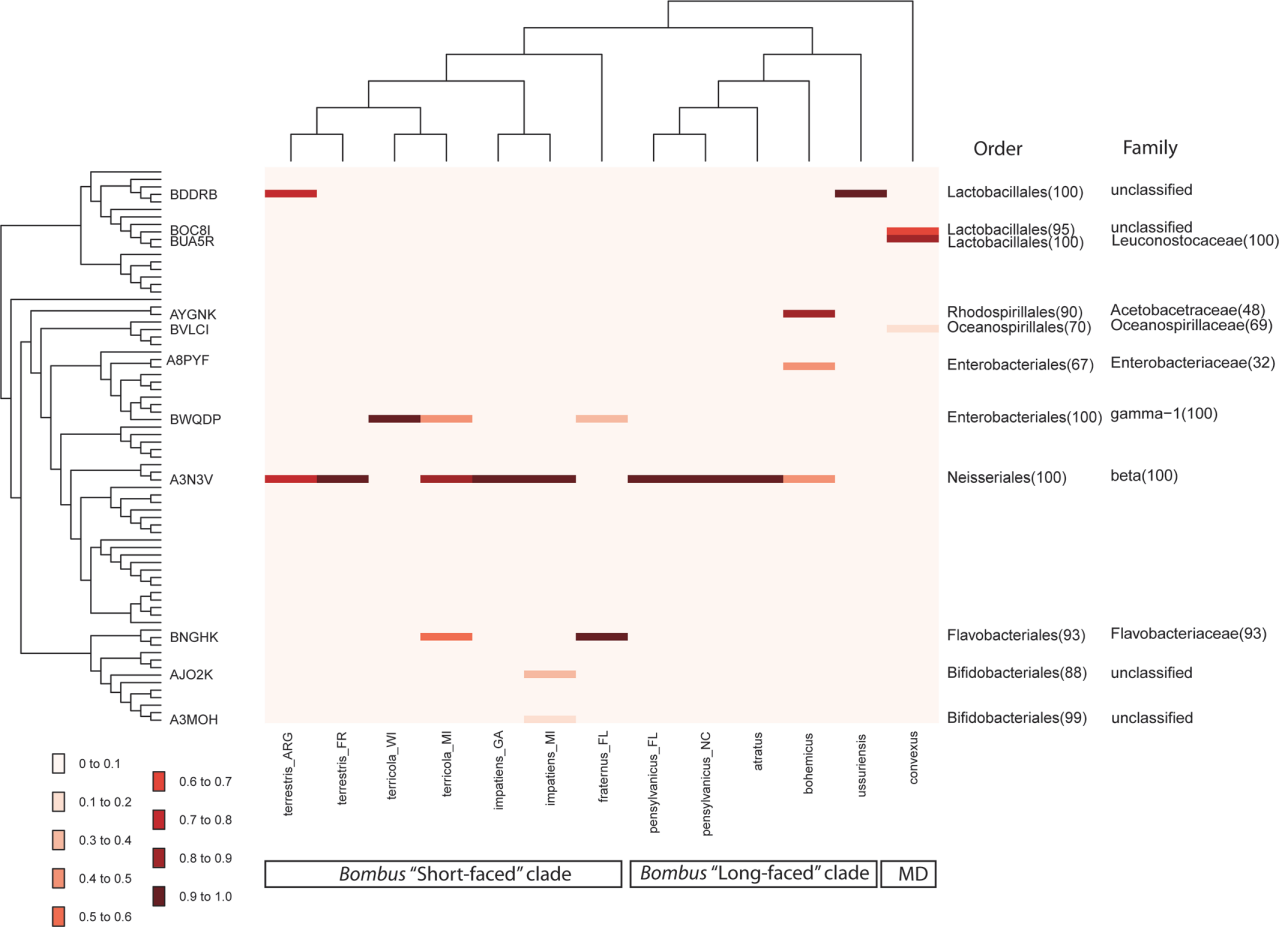
Heatmap showing relative abundance of each bacterial OTU (rows) in each of the pyrosequencing sample (columns). On the left is a cladogram showing phylogenetic relationships of the OTUs (tip labels = representative sequence ID). The IDs of the OTUs are abbreviated by only keeping their unique, last five characters (e.g., HUSOE0I01*BDDRB* to *BDDRB*). On the right are classifications of the OTUs at two taxonomic levels, if available. For clarity, only labels for OTUs with relative abundance > 10% are shown. Atop the heatmap, a cladogram indicates phylogenetic relationships of the bee species investigated. The bars at the bottom of the graph indicate the phylogenetic clade in which each bee species falls. MD = Mendacibombus (subgenus). See S4 Fig. for fully labeled tips.

For each pyrosequencing sample, we obtained 1,296 to 12,408 high-quality reads representing seven to 19 bacterial OTUs (Table 3). Sample-based rarefaction analysis showed that OTU richness curves largely plateaued. On the other hand, inter-sample rarefaction indicated that more bacterial OTUs are likely to be found with additional pyrosequencing samples (S3 Fig.). Three pyrosequencing samples, “*IMPATIENS_MI*”, “*PENSYLVANICUS_FL*” and “*TERRESTRIS_FR*”, had the highest estimated bacterial richness (10.4–11.9) when sequencing depth was normalized to that of “*BOHEMICUS*” (*n* = 1,296), the sample with the smallest number of reads (Table 3). While each pyrosequencing sample contained several OTUs (range: 7–19), many of the OTUs were rare (Fig. 2, S4 Fig.). In any particular sample, the maximum number of OTUs with relative abundance above 1% was five (average = 2.54 ± 1.20 [standard deviation, SE]) while the average number of OTUs with relative abundance above 5% was 1.92 ± 0.95 (S1 Table).

**Table 3:** Sequencing depth and number of OTUs detected for each pyrosequencing sample.

Pyrosequencing sample ID	*Bombus s*pecies	Number of sequences	Number of OTUs	Estimated number of OTU at 1,296 sequences (rarefaction based)
*ATRATUS*	*B*. *atratus*	9434	8	5.49
*BOHEMICUS*	*B*. *bohemicus*	1296	7	7.00
*CONVEXUS*	*B*. *convexus*	6982	8	5.93
*FRATERNUS_FL*	*B*. *fraternus*	11284	12	7.45
*IMPATIENS_GA*	*B*. *impatiens*	11556	9	5.48
*IMPATIENS_MI*	*B*. *impatiens*	4551	16	11.91
*PENSYLVANICUS_NC*	*B*. *pensylvanicus*	5005	15	8.46
*PENSYLVANICUS_FL*	*B*. *pensylvanicus*	12084	19	10.40
*TERRESTRIS_FR*	*B*. *terrestris*	5520	16	11.01
*TERRESTRIS_ARG*	*B*. *terrestris*	12408	13	7.47
*TERRICOLA_MI*	*B*. *terricola*	4616	10	7.96
*TERRICOLA_WI*	*B*. *terricola*	9227	11	6.08
*USSURENSIS*	*B*. *ussurensis*	12233	11	5.49

### Gut bacterial taxa

**Fig 3:**
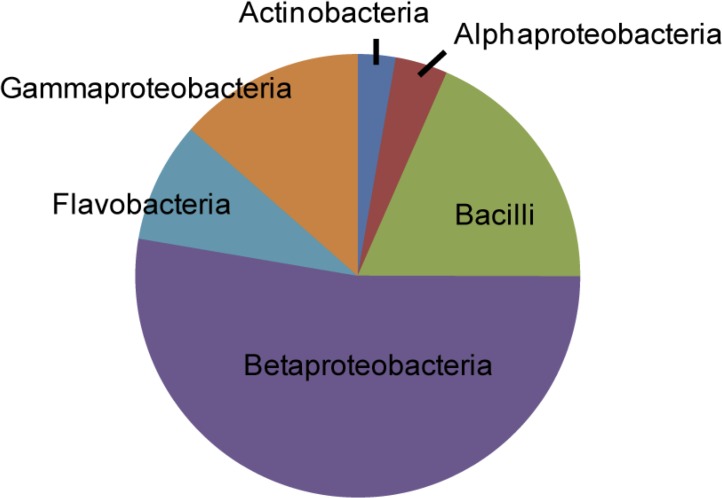
Relative abundance (averaged across 13 pyrosequencing samples) of different bacterial classes. The average number of sequences obtained from each pyrosequencing sample was 15,170.

Sequencing of all 13 pyrosequencing samples revealed bacteria from only six classes: Actinobacteria, Alphaproteobacteria, Bacilli, Betaproteobacteria, Flavobacteria and Gammaproteobacteria (Table 2). Overall, Betaproteobacteria was the most dominant class, accounting for 52.7 ± 42.2% of the bacterial sequences when abundance was averaged across 13 pyrosequencing samples. This was followed by Bacilli (18.5 ± 36.0%) and Gammaproteobacteria (13.5 ± 24.9%) (Fig. 3, S1 Table). The number of OTUs found in each class varied widely; it ranged from 20 each in Bacilli and Betaproteobacteria, to two in Alphaproteobacteria. Bacterial Orders containing OTUs with high abundance included Neisseriales (Betaproteobacteria), Lactobacillales (Bacilli) and Enterobacteriales (Gammaproteobacteria). At the opposite end of the spectrum, bacterial orders with OTUs with low overall abundance included Burkholderiales (Betaproteobacteria), Oceanospirillales (Gammaproteobacteria) and Bifidobacteriales (Actinobacteria) (Table 2).

Overall, only six bacterial OTUs from five different classes were found to be dominant across all 13 samples (i.e., OTUs possessing the highest relative read abundance in each pyrosequencing sample, range: 47.3%−99.3%). Based on BLASTn results, five of these OTUs have sequences that were highly similar (98.7%−100%) to named bacteria known either from the gut of bees, flowers or the immediate environment of bees (e.g., bee bread) (Table 4). The most prevalent of these dominant OTUs matched *Snodgrassella alvi*, found in 12 pyrosequencing samples. Further, it was the most dominant OTU in eight sequencing samples (Fig. 2, S4 Fig.). The other five dominant OTUs matched sequences of *Saccharibacter floricola*, *Fructobacillus tropaeoli*, *Lactobacillus kunkeei*, *Gilliamella apicola*, and an unnamed bacterium belonging to Family Flavobacteriaceae (Table 4). Other than the dominant OTUs, we uncovered some OTUs that were less dominant (rarely accounting for more than 5% of the reads in a sample), such as one classified as phylotype firm-5 (HUSOE0I01BP7S6, detected in seven bee species) and a Bifidobacteriales OTU (HUSOE0I01AJO2K, detected in five *Bombus* species) (S1 Table). At the level of individual pyrosequencing samples, the group of OTUs comprising phylotype beta (or *S*. *alvi*) appeared to be particularly diverse. Each sample that contained HUSOE0I01A3N3V (the most abundant of the beta/*S*. *alvi* OTUs) generally also contained at least two other closely related beta OTUs. Most of these rarer beta OTUs were unlikely to be variants caused by sequencing errors because they have non-trivial abundance (> 5 reads) and were found in more than one sample.

**Table 4:** Taxonomic assignments of six OTUs based on naïve Bayesian classification of 16S rRNA sequence.

Representative sequence (proceeded by HUSOE0I01)	Class-Order-Family (bootstrap confidence scores in parenthesis)	Number of samples in which OTU has highest relative abundance	Number of samples in which OTU was detected	Avg. relative abundance (%) of OTU across 13 samples	ID of GenBank match	% identity (length of query in bp)	Name of bacterium	Documented source of bacterium	References describing the species
AYGNK	Alphaproteobacteria (100)- Rhodospirillales (90)- Acetobacetraceae (48)	1	2	3.8	JF794031	98.9(445)	*Saccharibacter floricola*	Pollen	[57]
BUA5R	Bacilli (100)- Lactobacillales (100)- Leuconostocaceae (100)	1	4	3.9	AB542054	100.0(446)	*Fructobacillus tropaeoli*	Flower	[56]
BDDRB	Bacilli (100)- Lactobacillales (100)- unclassified	1	5	11.4	KF600556	100.0(446)	*Lactobacillus kunkeei*	Gut of *Apis mellifera*, nectar, honey and beebread	[58]
A3N3V	Betaproteobacteria (100)- Neisseriales (100)- beta (100)	8	12	52.1	JQ746648	98.7(445)	*Snodgrassella alvi*	Gut of *Bombus vagans*	[76]
BNGHK	Flavobacteria (93)- Flavobacteriales (93)- Flavobacteriaceae (93)	1	7	8.8	DQ837639	99.1(443)	unnamed	Gut of *Apis mellifera*	NA
BWQDP	Gammaproteobacteria (100)- Enterobacteriales (100)- gamma-1 (100)	1	9	10.3	JQ936675	99.8(445)	*Gilliamella apicola*	Gut of *Bombus bimaculatus*	[76]

In parentheses are bootstrap confidence of each assignment. Each of these OTUs was the most dominant OTU in at least one pyrosequencing sample. Also included are information related to each OTUs closest matching GenBank sequence reported by a published paper.

### Community distinctiveness based on phylogenetic distances among bacteria

**Fig 4:**
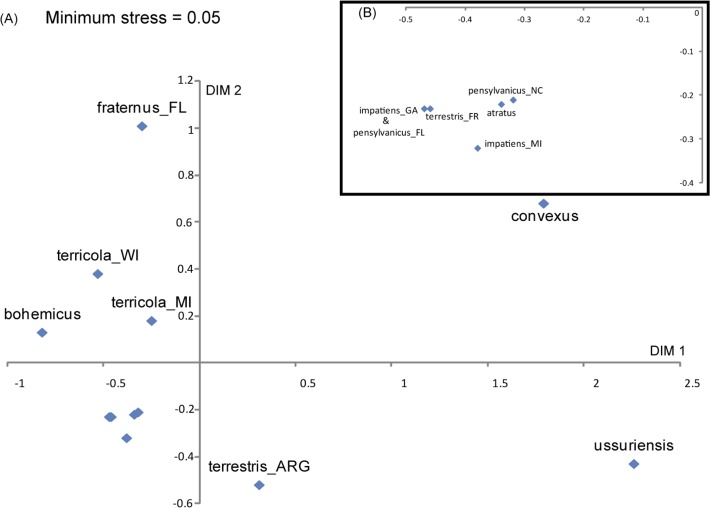
Plot of pyrosequencing samples in the 2-D nMDS space based on inter-sample UniFrac distances (A). Minimum stress after 999 iterations is 0.05. The cluster of samples in the lower-left quadrate (ones without labels) is expanded in the top right corner (B).

Based on the UniFrac “all environments together” test, there was an overall significant difference among the pyrosequencing samples, a difference that was based on a combination of their community composition and the phylogenetic distances among the OTUs within the communities (*P* ≤ 0.01, after correcting for multiple comparisons). This difference was primarily driven by pyrosequencing samples “*FRATERNUS_FL*”, “*USSURENSIS*” and “*CONVEXUS*” (Table 5). The distinctiveness of these microbial communities was supported by NMDS and jackknife hierarchical clustering (Fig. 4 and S5 Fig.). The sample “*CONVEXUS*” was dominated by three bacterial OTUs, all of which were either absent or present at very low abundance in other pyrosequencing samples. These three OTUs included two closely related taxa belonging to the order of Lactobacillales (relative abundance = 50.3% and 37.5%, respectively) and one belonging to Oceanospirillales (11.2%) (Fig. 2, S4 Fig.). The sample “*USSURENSIS*” was dominated by a member of Lactobacillales (relative abundance = 99.3%), which occurred in significant abundance in only one other pyrosequencing sample (“*TERRESTRIS_ARG*”). The third highly distinctive pyrosequencing sample was “*FRATERNUS_FL*”. It contained two principal OTUs, belonging to Flavobacteriales (76.2%) and Enterobacteriales (17.1%), respectively. Both of these OTUs occurred in significant abundance in only 1–2 other gut bacterial communities (Fig. 2, S4 Fig.). The communities of five pyrosequencing samples (“*TERRESTRIS_FR*”, “*IMPATIENS_GA*”, “*PENSYLVANICUS_FL*”, “*PENSYLVANICUS_NC*”, and “*ATRATUS*”) were highly similar; they were all dominated (relative abundance > 94%) by an OTU whose representative sequence (HUSOE0I01A3N3V) closely matched that of *S*. *alvi* (see **Gut bacterial taxa)**.

**Table 5:** Significance of UniFrac tests that compared a single pyrosequencing sample against all other samples combined.

Pyrosequencing sample	*P*-value
*ATRATUS*	1.00
*BOHEMICUS*	0.40
*CONVEXUS*	**0.02**
*FRATERNUS_FL*	**0.05**
*IMPATIENS_GA*	0.63
*IMPATIENS_MI*	0.12
*PENSYLVANICUS_FL*	0.17
*PENSYLVANICUS_NC*	0.38
*TERRESTRIS_ARG*	0.50
*TERRESTRIS_FR*	0.67
*TERRICOLA_MI*	0.73
*TERRICOLA_WI*	0.61
*USSURENSIS*	**0.02**

A significant *P-*value (*P* < 0.05, in bold) indicates that the bacterial community tested has more unique branch length than expected by chance.

### Effects of species and locality on gut microbiota structure

**Fig 5:**
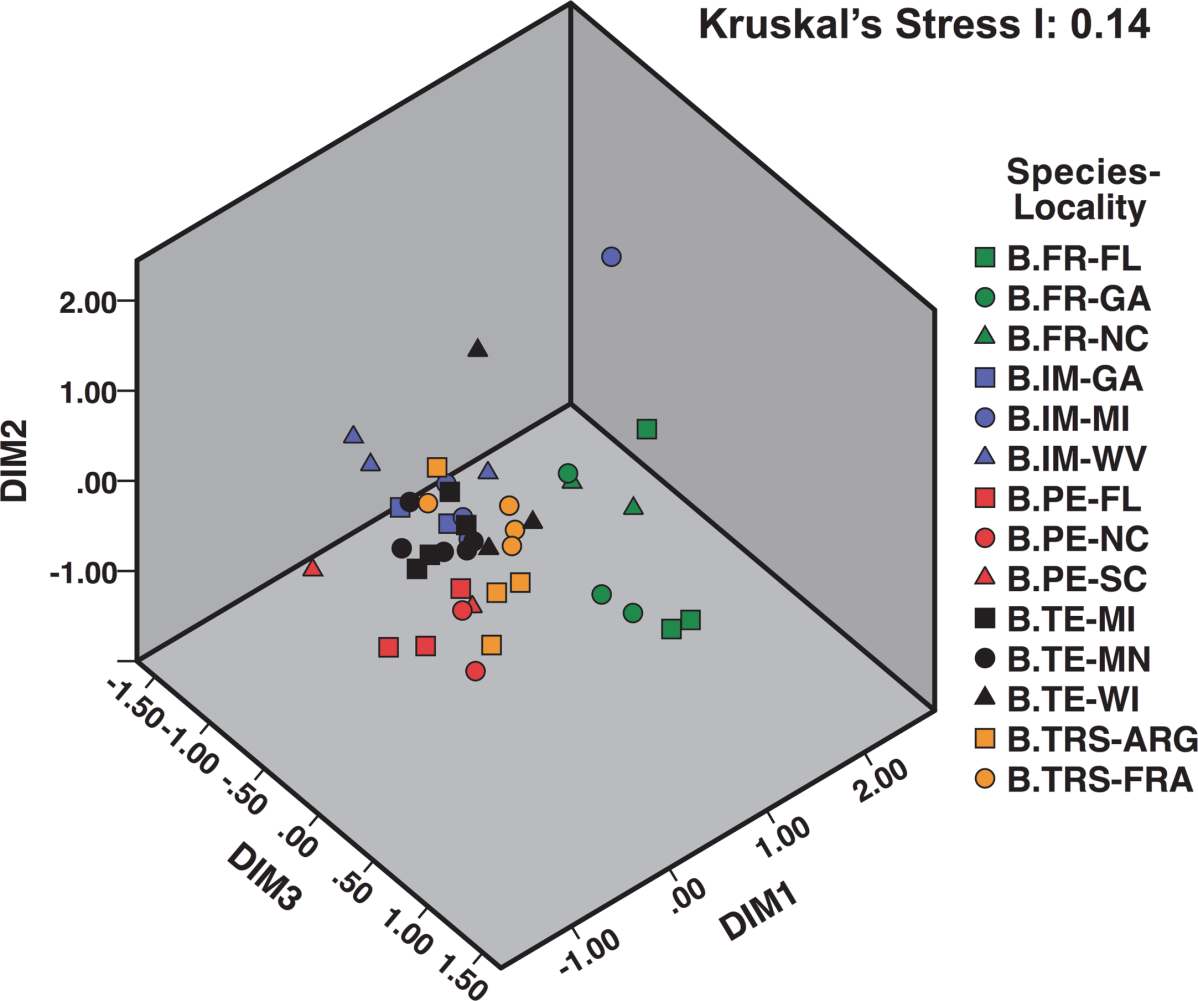
Three-dimensional (DIM1–3) nMDS ordination of ARISA profiles of different bumble bee gut DNA samples. Corresponding bumble bee species and populations (collection sites) are illustrated by the colors and shapes of the markers on the right. Abbreviations: B.FR = *B*. *fraternus*; B.IM = *B*. *impatiens*; B.PE = *B*. *pensylvanicus*; B.TE = *B*. *terricola*; B.TRS = *B*. *terrestris*. Abbreviations following species names represent different collection sites, sorted by states in the US (FL, GA, MI, MN, NC, SC, WI, and WV), or across countries (ARG and FRA). ARG: Argentina, FRA: France.

After noise-filtering of the ARISA data, we observed an average of 125 fragments per sample. A 3-D plot of the NMDS analysis based on Bray-Curtis dissimilarity measures showed that samples tended to cluster by bumble bee species, and by locations within species (Fig. 5). The plot also showed trends resembling that of the 2-D NMDS plot based on the sequencing data (Fig. 4); microbial communities of *B*. *fraternus* were relatively distinct from those of the other four *Bombus* species analyzed (*B*. *pensylvanicus*, *B*. *terricola*, *B*. *terrestris*, and *B*. *impatiens*). A two-way unbalanced PERMANOVA including “species” (fixed effect) and “locality” (random effect nested in species) showed that both factors significantly influenced the gut microbiota structures of the bees examined (Species, *P* = 0.0001; Locality, *P* = 0.0001).

### Microscopy results

**Fig 6:**
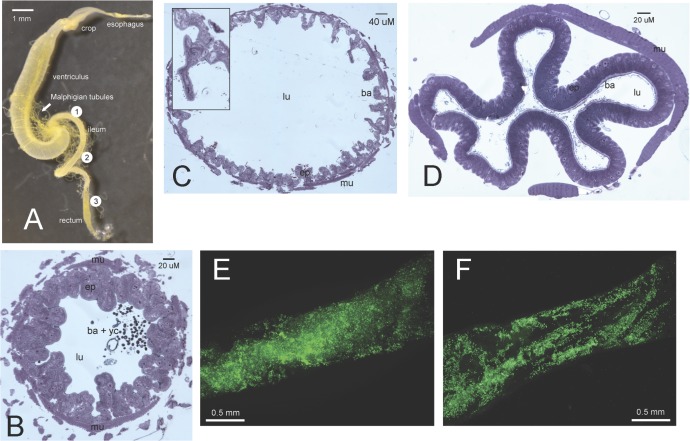
Images of *B. impatiens* gut (A) from light (B, C and D) and fluorescent microscopy (E and F). (A) shows the whole alimentary canal and general locations where sectioning and imaging were made (multiple individuals were used to create the dissected gut and microscopy images). Section made at (1), (2) and (3) were used to produce images (B), (C) and (D), respectively. Fluorescent images (E) and (F) were produced by visualizing a portion of the ileum located between (1) and (2), and between (2) and (3), respectively. (B) shows both yeast and bacterial cells in the lumen, whereas (C) and (D) show only bacterial cells in the lumen. Villi and longitudinal folds composed of epithelial cells can be observed in (C) and (D), respectively. Insert in (C) shows a villus and bacterial cells (black dots) in greater detail. In (E) and (F), each fluorescing green spot represents one bacterial cell. Abbreviations: ba = bacteria; ep = epithelial cells; lu = lumen; mu = transverse muscle; yc = yeast cell.

In *B*. *impatiens*, we observed through light microscopy both yeast and bacterial cells in the lumen of the anterior part of the ileum (Fig. 6B). In the middle and posterior sections of the ileum, bacteria alone were found in the lumen (Fig. 6C and 6D). Based on fluorescent microscopy, these bacteria appeared to form a biofilm on the cuticular intima, a lining separating the lumen from the epithelial cells (Fig. 6E, F). Because of the presence of longitudinal folds in the posterior portion of the ileum, the distribution of bacteria in the posterior portion appeared to be less even when compared to the anterior portion.

## Discussion

Using Roche 454 pyrosequencing, we conducted deep sampling to characterize the gut microbiota of bumble bee species from multiple subgenera. Classification of the 74 bacterial OTUs included bacterial families that were consistent with other reports on gut microbes of hymenopteran insects [15,23], suggesting good classification results using our customized training set. Regardless of the bumble bee species, the gut bacterial communities were not taxonomically diverse, with only 1–3 common (relative abundance > 5%) bacterial OTUs within the each microbiota. Among the 74 bacterial OTUs we delimited, the two most widespread and abundant bacteria (*S*. *alvi* and *G*. *apicola*) were also identified as core gut bacterial species in previous studies of honey bees and several bumble bee species [15,33]. The detection of these two bacterial species in most of our pyrosequencing samples, including many previously unexamined bumble bee species spanning large phylogenetic distances, suggests they play important functional roles in bees. These two bacterial species also have been shown to exhibit co-phylogenetic patterns with their hosts [32], suggesting long-term coevolution with their hosts and a tendency for vertical transmission.

Of the remaining four dominant OTUs, three have high (> 98.9%) 16S rRNA sequence similarity to previously named bacterial species: *S*. *floricola*, *F*. *tropaeoli* and *L*. *kunkeei*. These three bacteria are aerotolerant, osmophilic/osmotolerant lactic acid or acetic acid bacteria that, unlike *S*. *alvi* and *G*. *apicola*, were discovered and described from environments associated with bees, including flowers, hive, bee bread (processed, packed pollens) and grape juice [56–59]. This suggests that some of the gut bacteria have greater physiological tolerance for living outside the gut and may be amenable to environmental transmission. Our ARISA results showing that not only species but also geographic location of the host affects its gut bacterial community, again supports existing evidence that at least some members of the gut microbial community appear to be environmentally transmitted [60–63]. This may explain the relatively different gut microbial communities found between populations of same *Bombus* species collected in different geographic regions, such as the microbiota of *B*. *terrestris* from France and Argentina (Fig. 4 and S5). European colonies of *B*. *terrestris* were shipped to South America for commercial pollination and became naturalized since the 1990s. The species then spread to Argentina, and may have been exposed to environmental resources considerably different to those in Europe [64,65].

In identifying common bacteria found in the gut of bumble bees, we can rely on previously conducted *in vivo* and *in vitro* studies (often focusing on related bacteria in honey bees) to infer the roles they play in contributing to colony and individual health. Outside of the gut, bacteria such as *L*. *kunkeei* and *S*. *floricola* may benefit bumble bees by producing weak acids that aid in the fermentation and preservation of stored pollens in the nest [66,67]. The gut is likely to be an important source of these bacteria because worker bumble bees first moisten collected pollen with regurgitated nectar before packing it onto the hind leg pollen-carrying structure (corbicula) [59,68]. In the ileum, bacteria may enhance nutrient absorption by helping to break down food molecules. This is supported by a functional genomics study that found that the gut microbiome of honey bees (which share a number of gut bacterial species with bumble bees) was enriched for genes associated with carbohydrate breakdown (including those involved in pectin breakdown) and transport [17]. In addition to nutrition, evidence is mounting that gut bacteria benefit their bee hosts by contributing to the latter’s defenses against non-native bacteria and protistan enemies. For example, Vasquez et al. [19] found that lactic acid bacteria (*Lactobacillus* spp. and *Bifidobacterium* spp.) isolated from honey bees have inhibition properties on microorganisms such as yeasts and other bacteria (including *Melissococcus plutonius*, the causative agent of European foulbrood), with *L*. *kunkeei* (dominant in “*USSURENSIS*” and “*TERRESTRIS_ARG*”) showing the strongest effects. Similarly, studies have shown that bacteria cultures from honey bee crops or larvae inhibited the growth of bacterial pathogens of bees [69,70]. As one of the few studies that focused on bumble bee protistan parasites, Koch and Schmid-Hempel [12] showed that bees first treated with antibiotics then subsequently fed with feces from nest mates have lower parasite load (Trypanosomatidae: *Crithidia bombi*) compared to bees not given viable bacterial inoculation. While the study was groundbreaking, the mechanisms by which gut bacteria exert their protective functions against enteric protists are not entirely clear. Similar to honeybees, we found that bacteria in the ileum of the bumble bee species *B*. *impatiens* forms biofilms on the inside surface of the gut epithelium (Fig. 6). This suggests that bumble bee gut microbiotas may have specific functions in their hosts, such as providing nutritional benefits or defense against intestinal pathogens. Future research in these areas will have important implications for the conservation of corbiculate bees worldwide, as numerous species and populations are purportedly threatened by invasive parasites or deficient nutrition [71–73].

In addition to the dominant bacterial OTUs, we also uncovered numerous rare ones. It is possible that these could be the result of sequencing errors despite our stringent computational pipeline, removal of singletons and the clustering of sequences into OTUs. The inflation of microbial diversity as a result of sequencing errors is a recognized methodological artifact of next-generation sequencing [74]. Nonetheless, several of the rare OTU appear authentic and likely have important functions because of their ubiquity or moderate abundance in a small number of samples. Rare OTUs classified under beta/*S*. *alvi* are particularly interesting because a number of them always coexist with the dominant beta/*S*. *alvi* OTU when the latter is present. A similar situation was reported in honey bees in which a number of closely related *S*. *alvi* genomes coexisted at the level of colony, if not at the level of individual bees [17]. Given that gene content of bacteria can differ significantly even when their 16S rRNA sequences are very similar [75], these minor beta/*S*. *alvi* variants may play complementary functional roles to the dominant bacterial strains.

## Supporting Information

S1 Fig Phylograms (Bayesian consensus trees) based on 16S rRNA alignment. The trees show classifications (A—class, B—order, C—family) of representative sequences of 74 bacterial OTUs delimited in this study. Numbers in parentheses next to names indicate bootstrap confidence scores of the classifications.
(TIF)

S2 Fig Consensus Bayesian inference phylogenetic tree based on a bacterial 16S rRNA alignment. The tree comprises: 1) 274 full or almost full-length sequences from a previous work (7) and 2) 74 sequences representing 74 bacterial OTUs (this study). The 274 sequences (NR) are part of a training set that also contains sequences from the SILVA database. Tip labels for the trees denote: (A) sequence ID and (B-D) taxonomic information at the class, order and family levels. Taxonomic information is either provided (NR sequences) or generated using a naïve Bayesian classifier (this study, all sequence ID’s start with “HUSOE”). Taxonomic information may be missing for some NR sequences at lower taxonomic levels. Sequences from this study may be “unclassified” because of missing taxonomic information in the training set. In (A), node labels represent Bayesian posterior probabilities of nodes. For clarity, labels for some nodes closer to the tips are not shown. Sequences with a star (sequences from this study, n = 6) or circle (NR sequences, n = 1) have mismatches between their placement on the phylogenetic tree and their taxonomic classification at one or more levels. For sequences generated in this study, numbers in parentheses represent bootstrap confidence scores for taxonomic classification (B-D). Tree is midpoint rooted.
(PDF)

S3 Fig Rarefaction curves showing saturation of sampling at the level of reads and pyrosequencing samples. (A) Individual rarefaction curves of 13 pyrosequencing samples showing estimated number of OTUs detected for each additional 100 sequences. (B) Rarefaction curve showing the number of distinct OTUs discovered as more sequencing samples were added. Error bars depict 95% confidence intervals.
(PDF)

S4 Fig Heatmap showing relative abundance of each bacterial OTU (rows) in each of the sequencing samples (columns). On the left is a cladogram showing phylogenetic relationships of the OTUs (tip labels = representative sequence ID). On the right are classifications of the OTUs at two taxonomic levels (left = order, right = family), if available. Atop the heatmap, a cladogram indicates phylogenetic relationships of the bee species investigated.
(PDF)

S5 Fig Hierarchical clustering of bumble bee gut microbial community profiles. The clustering is based on the profiles’ normalized weighted inter-sample unique fraction (UniFrac) distances derived from pyrosequencing data. Jacknife resampling was carried out (values next to each branch) to assess the robustness of the cluster analysis.
(PDF)

S1 File Supplemental Methods. (DOCX)

S1 Table Taxonomic classification of representative sequences of 74 OTUs obtained from 16S rRNA amplicon sequencing of 13 pyrosequencing samples. Also provided are number of sequences that belong to each OTU. Numbers in parenthesis next to each taxonmic classification represents bootstrap confidence.
(PDF)
