# Supplementary figures and images for: Deep Sequencing and Ecological Characterization of Gut Microbial Communities of Diverse Bumble Bee Species
Source: PLoS One. 2015 Mar 13;10(3):e0118566. doi: 10.1371/journal.pone.0118566 (PMC4359114; doi:10.1371/journal.pone.0118566)

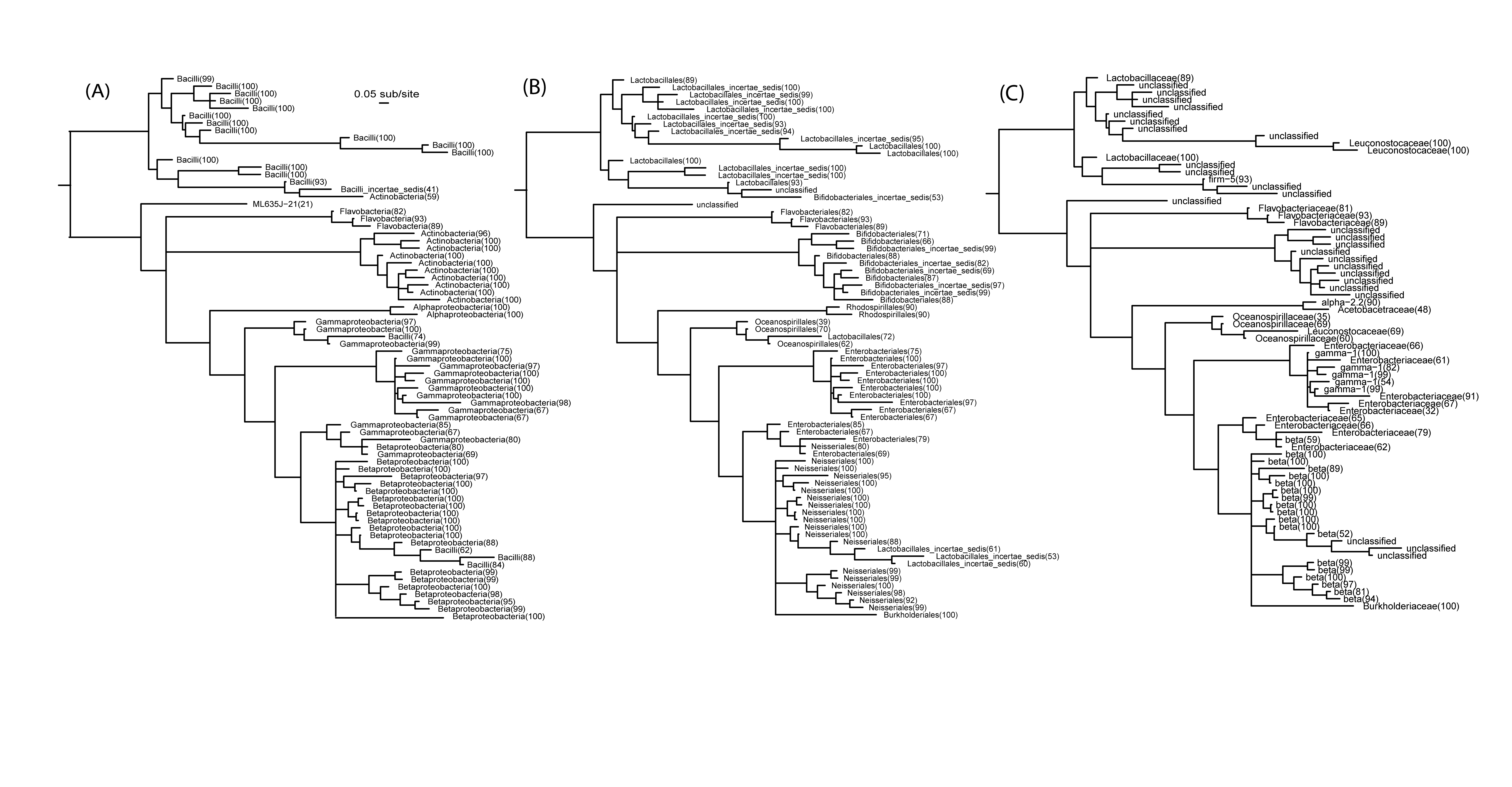

Supplement: S1 Fig — The trees show classifications (A—class, B—order, C—family) of representative sequences of 74 bacterial OTUs delimited in this study. Numbers in parentheses next to names indicate bootstrap confidence scores of the classifications. (TIF) [file pone.0118566.s001.tif]

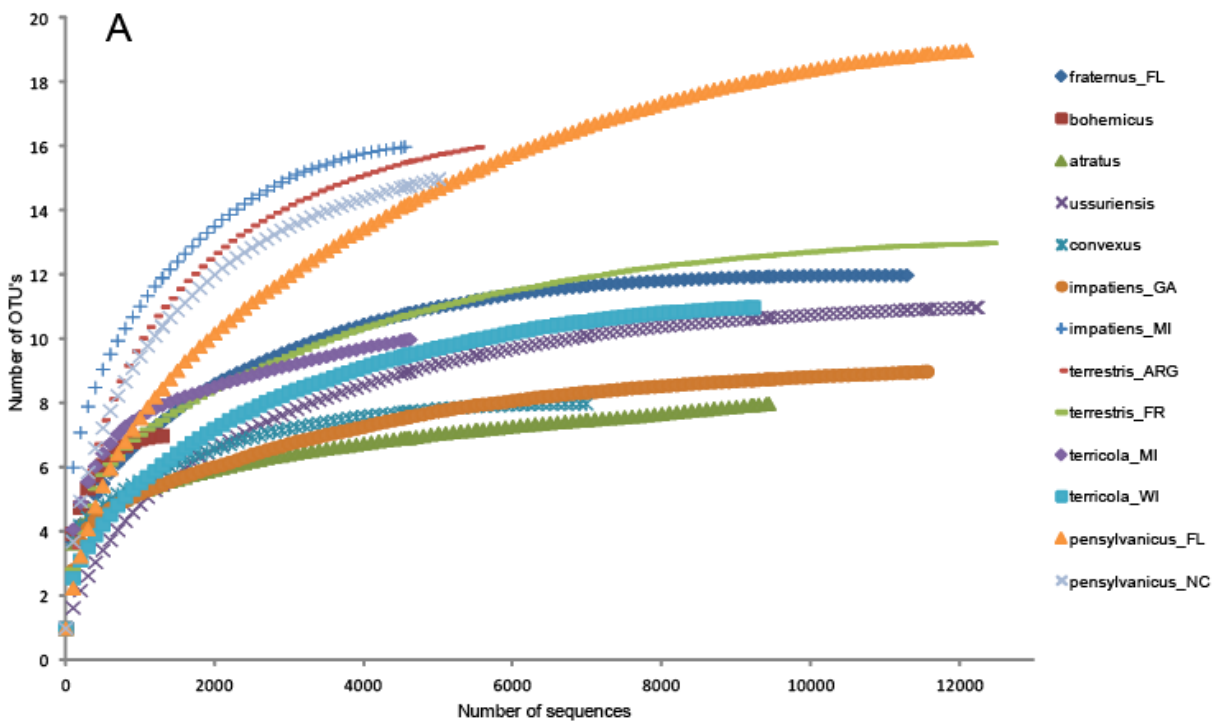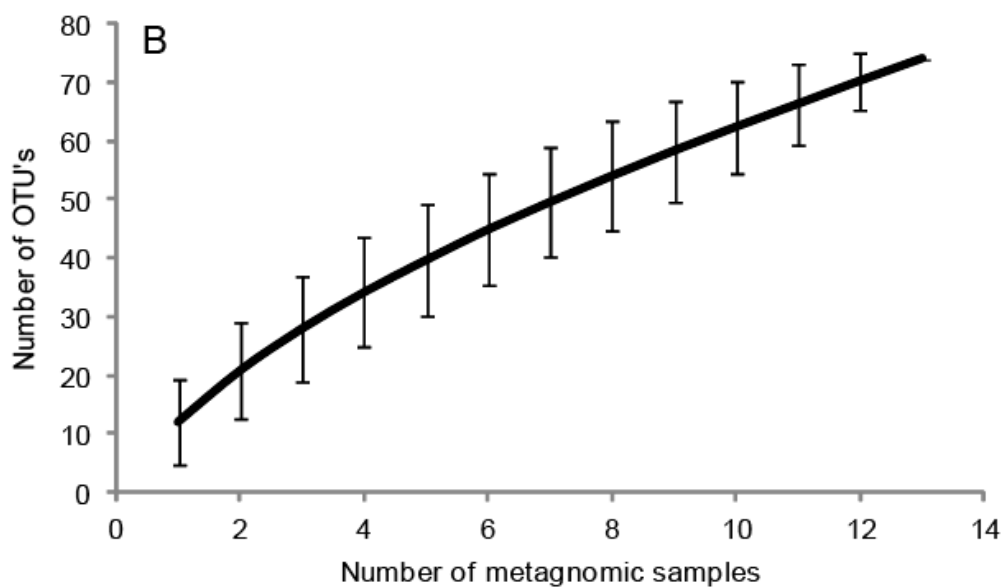

Supplement: S3 Fig — (A) Individual rarefaction curves of 13 pyrosequencing samples showing estimated number of OTUs detected for each additional 100 sequences. (B) Rarefaction curve showing the number of distinct OTUs discovered as more sequencing samples were added. Error bars depict 95% confidence intervals. (PDF) [file pone.0118566.s003.pdf]

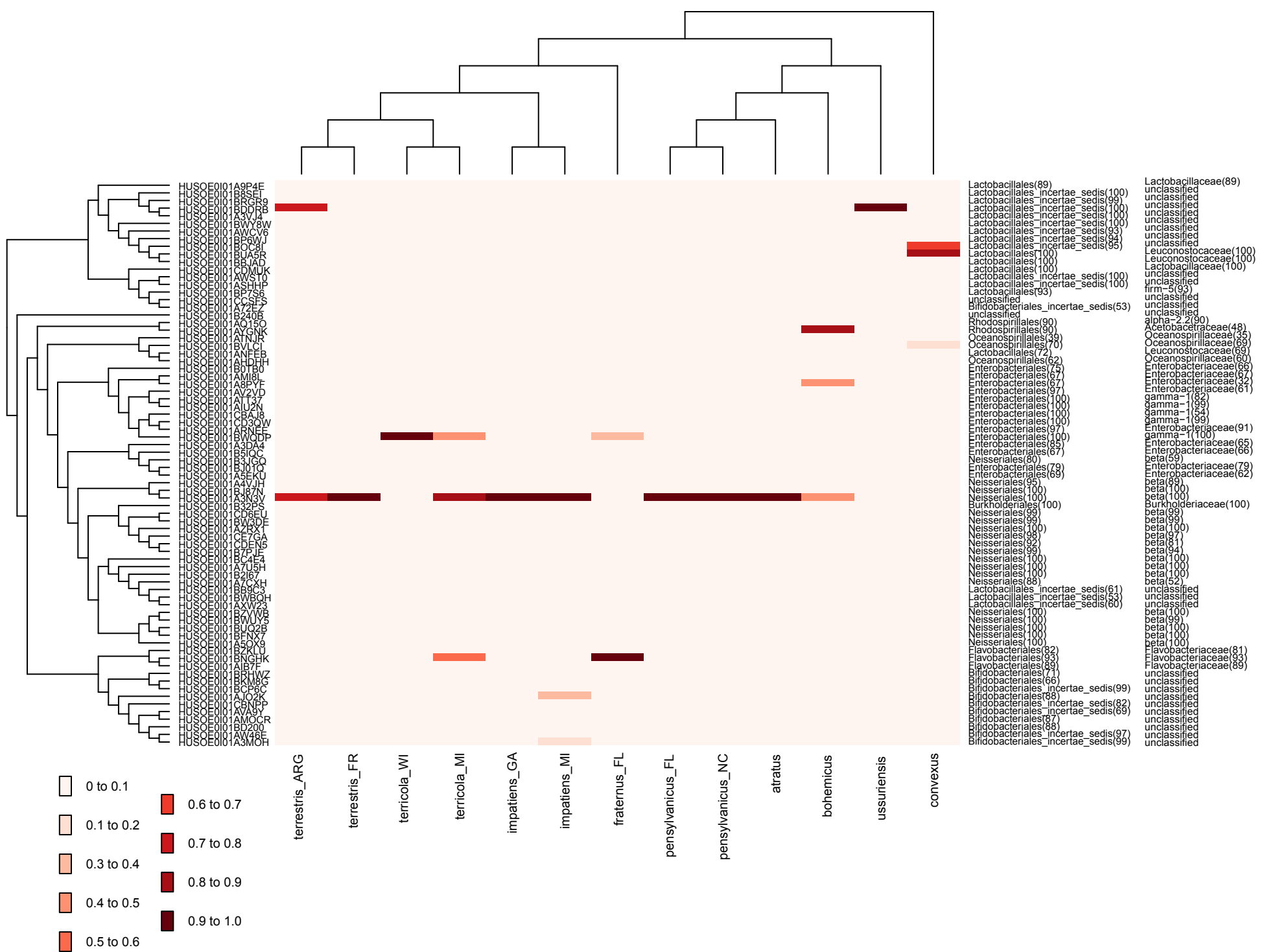

Supplement: S4 Fig — On the left is a cladogram showing phylogenetic relationships of the OTUs (tip labels = representative sequence ID). On the right are classifications of the OTUs at two taxonomic levels (left = order, right = family), if available. Atop the heatmap, a cladogram indicates phylogenetic relationships of the bee species investigated. (PDF) [file pone.0118566.s004.pdf]

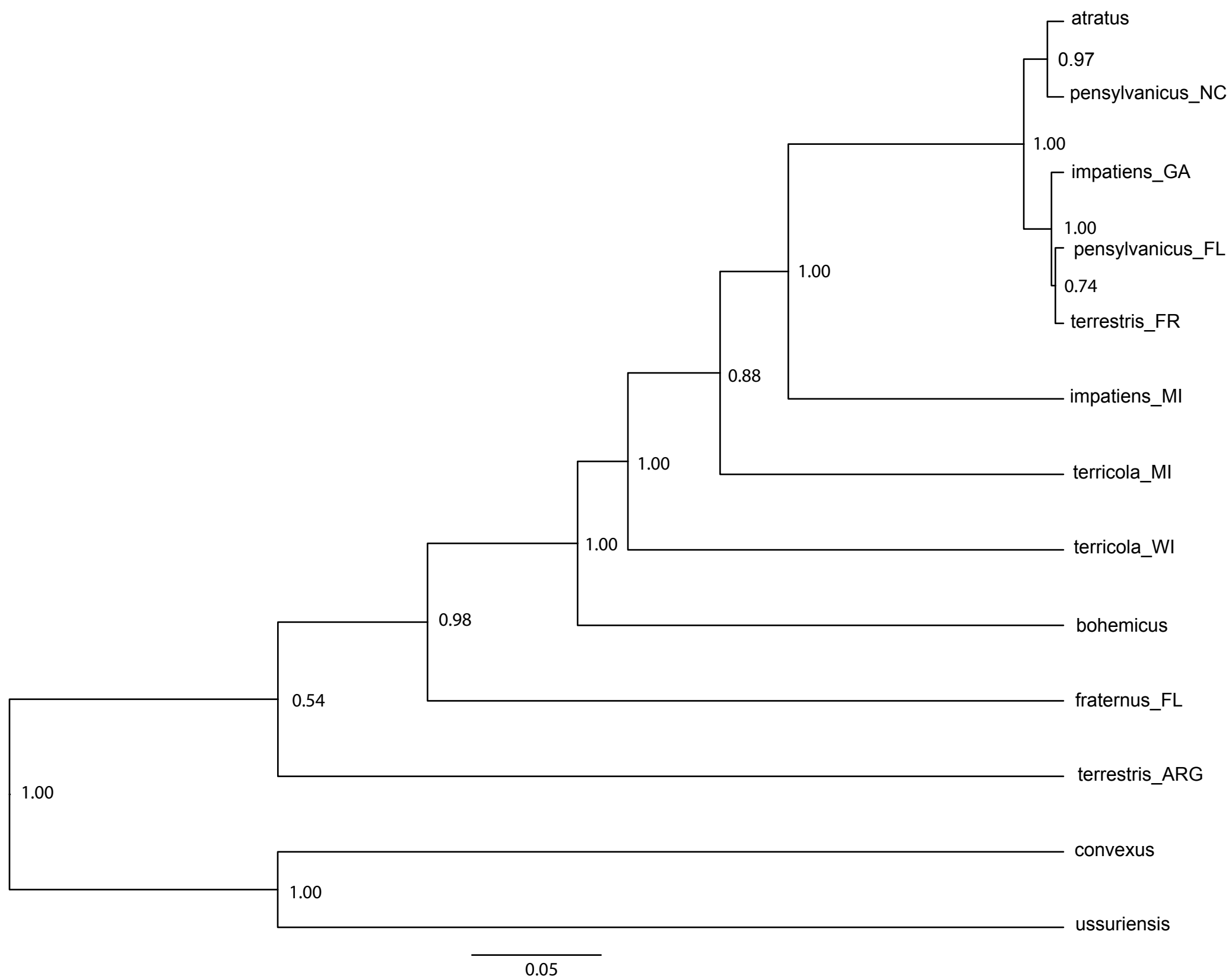

Supplement: S5 Fig — The clustering is based on the profiles’ normalized weighted inter-sample unique fraction (UniFrac) distances derived from pyrosequencing data. Jacknife resampling was carried out (values next to each branch) to assess the robustness of the cluster analysis. (PDF) [file pone.0118566.s005.pdf]
