# Supplementary material for: Deep Sequencing and Ecological Characterization of Gut Microbial Communities of Diverse Bumble Bee Species
Source: PLoS One. 2015 Mar 13;10(3):e0118566. doi: 10.1371/journal.pone.0118566 (PMC4359114; doi:10.1371/journal.pone.0118566)
Supplement: S2 Fig — The tree comprises: 1) 274 full or almost full-length sequences from a previous work (7) and 2) 74 sequences representing 74 bacterial OTUs (this study). The 274 sequences (NR) are part of a training set that also contains sequences from the SILVA database. Tip labels for the trees denote: (A) sequence ID and (B-D) taxonomic information at the class, order and family levels. Taxonomic information is either provided (NR sequences) or generated using a naïve Bayesian classifier (this study, all sequence ID’s start with “HUSOE”). Taxonomic information may be missing for some NR sequences at lower taxonomic levels. Sequences from this study may be “unclassified” because of missing taxonomic information in the training set. In (A), node labels represent Bayesian posterior probabilities of nodes. For clarity, labels for some nodes closer to the tips are not shown. Sequences with a star (sequences from this study, n = 6) or circle (NR sequences, n = 1) have mismatches between their placement on the phylogenetic tree and their taxonomic classification at one or more levels. For sequences generated in this study, numbers in parentheses represent bootstrap confidence scores for taxonomic classification (B-D). Tree is midpoint rooted. (PDF) [file pone.0118566.s002.pdf]

(A)

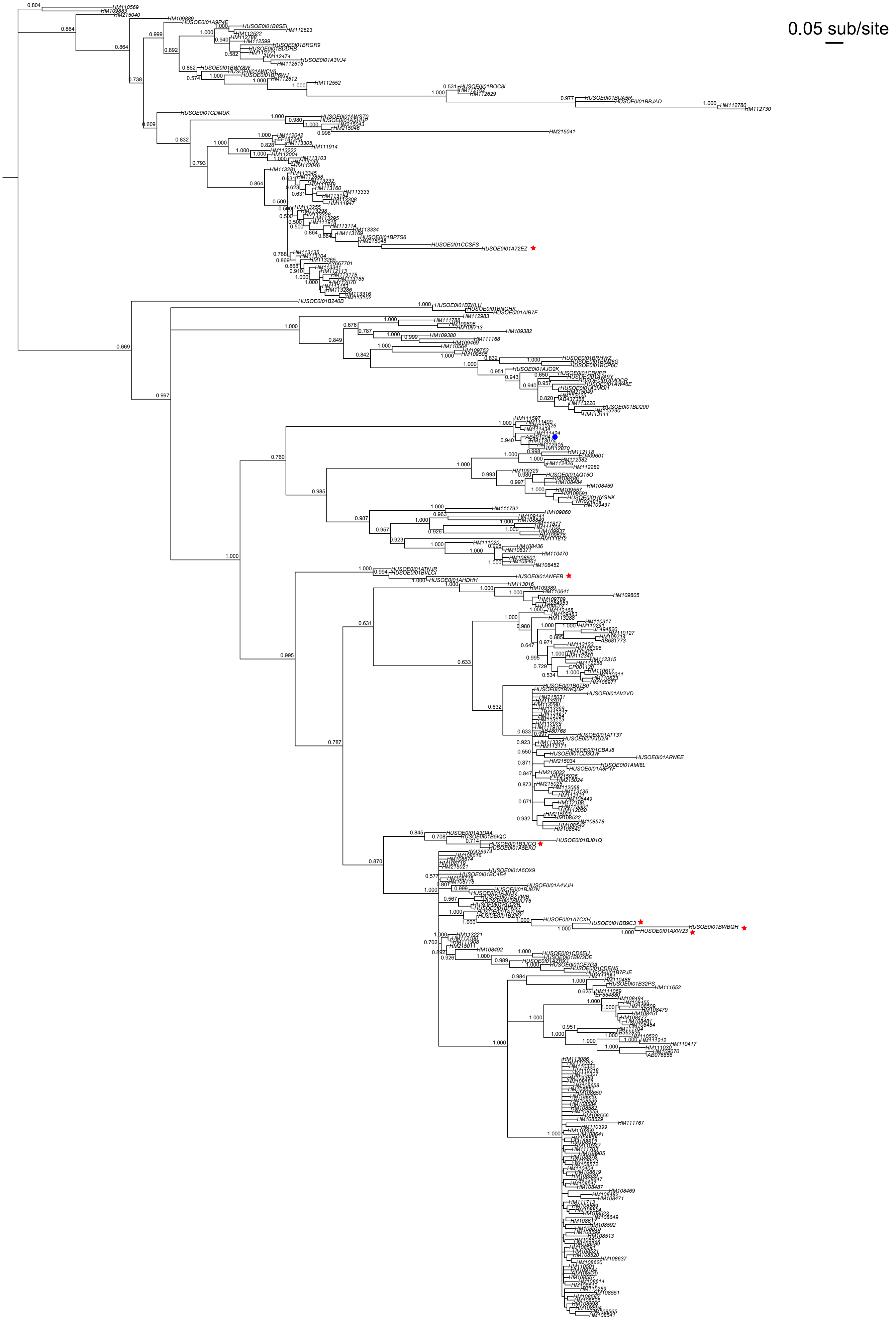

(B)

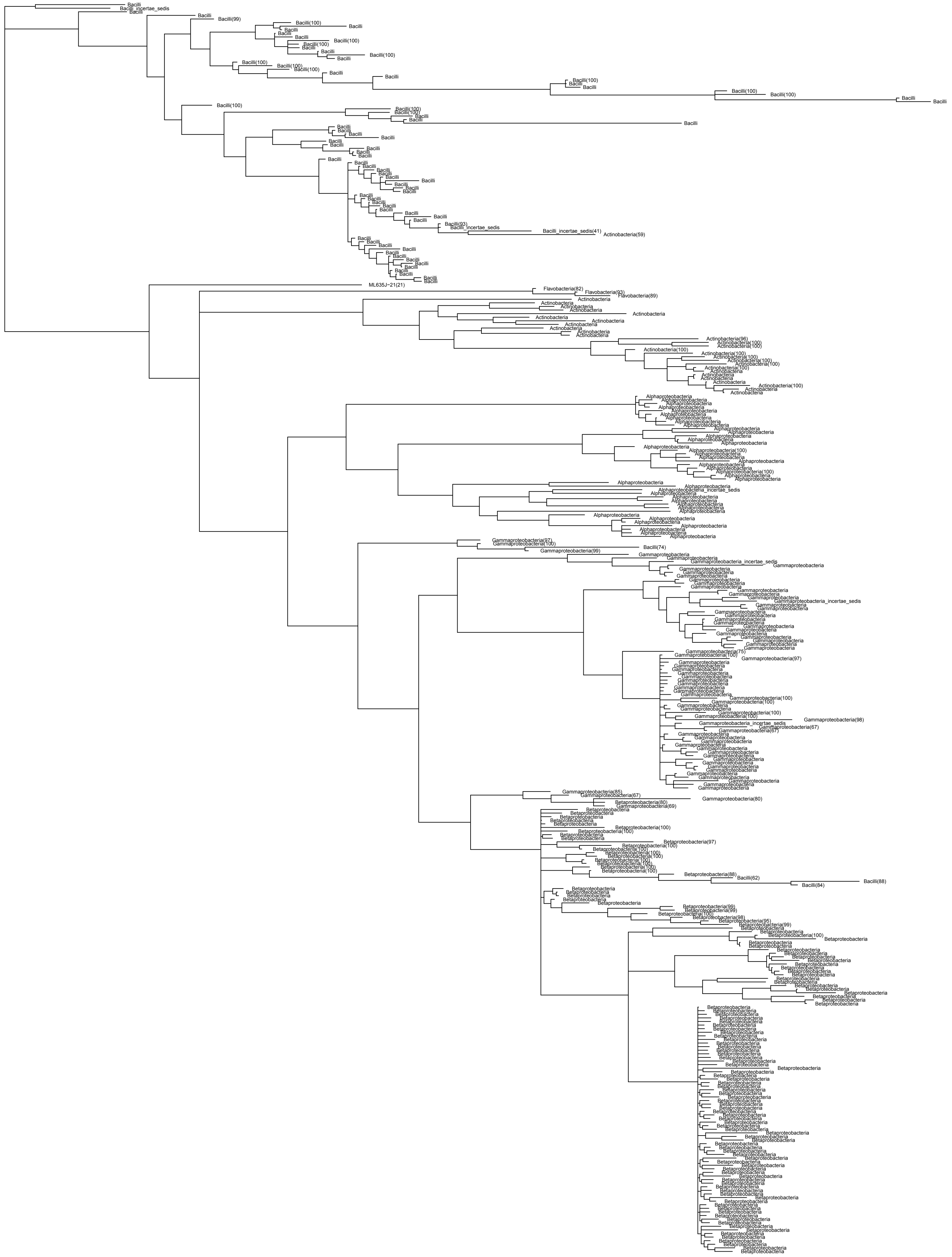
Bacilli
Bacilli_incertae_sedis
Bacilli(99)
Bacilli(100)
Bacilli(93)
Bacilli_incertae_sedis(41)
Actinobacteria(59)
ML635J−21(21)
Flavobacteria(82)
Flavobacteria(93)
Flavobacteria(89)
Actinobacteria
Actinobacteria(96)
Actinobacteria(100)
Alphaproteobacteria
Alphaproteobacteria(100)
Alphaproteobacteria_incertae_sedis
Gammaproteobacteria(97)
Gammaproteobacteria(100)
Bacilli(74)
Gammaproteobacteria(99)
Gammaproteobacteria
Gammaproteobacteria_incertae_sedis
Gammaproteobacteria(75)
Gammaproteobacteria(98)
Gammaproteobacteria(67)
Gammaproteobacteria(85)
Gammaproteobacteria(80)
Betaproteobacteria(80)
Gammaproteobacteria(69)
Betaproteobacteria
Betaproteobacteria(100)
Betaproteobacteria(97)
Betaproteobacteria(88)
Bacilli(62)
Bacilli(88)
Bacilli(84)
Betaproteobacteria(99)
Betaproteobacteria(98)
Betaproteobacteria(95)

(C)

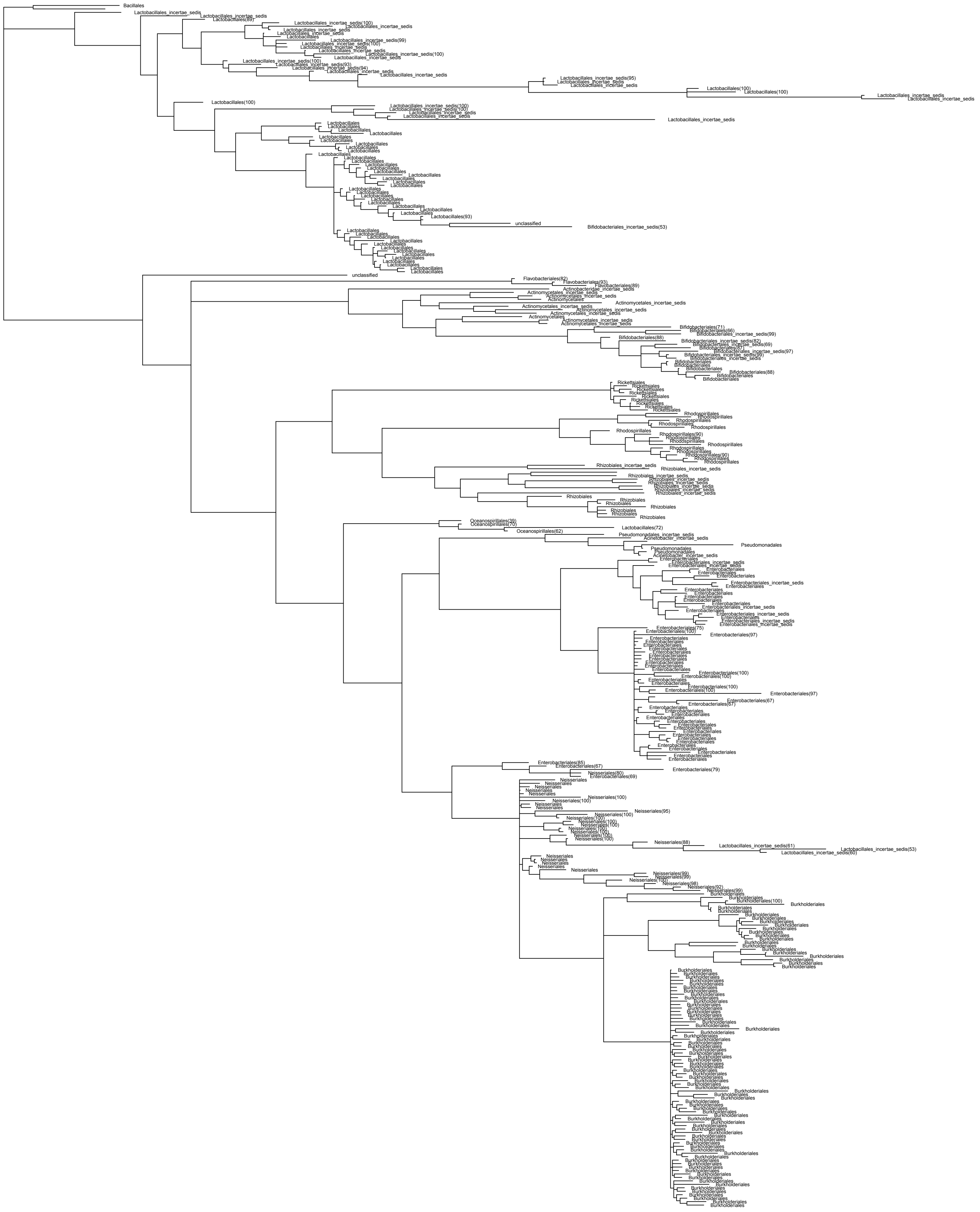

(D)

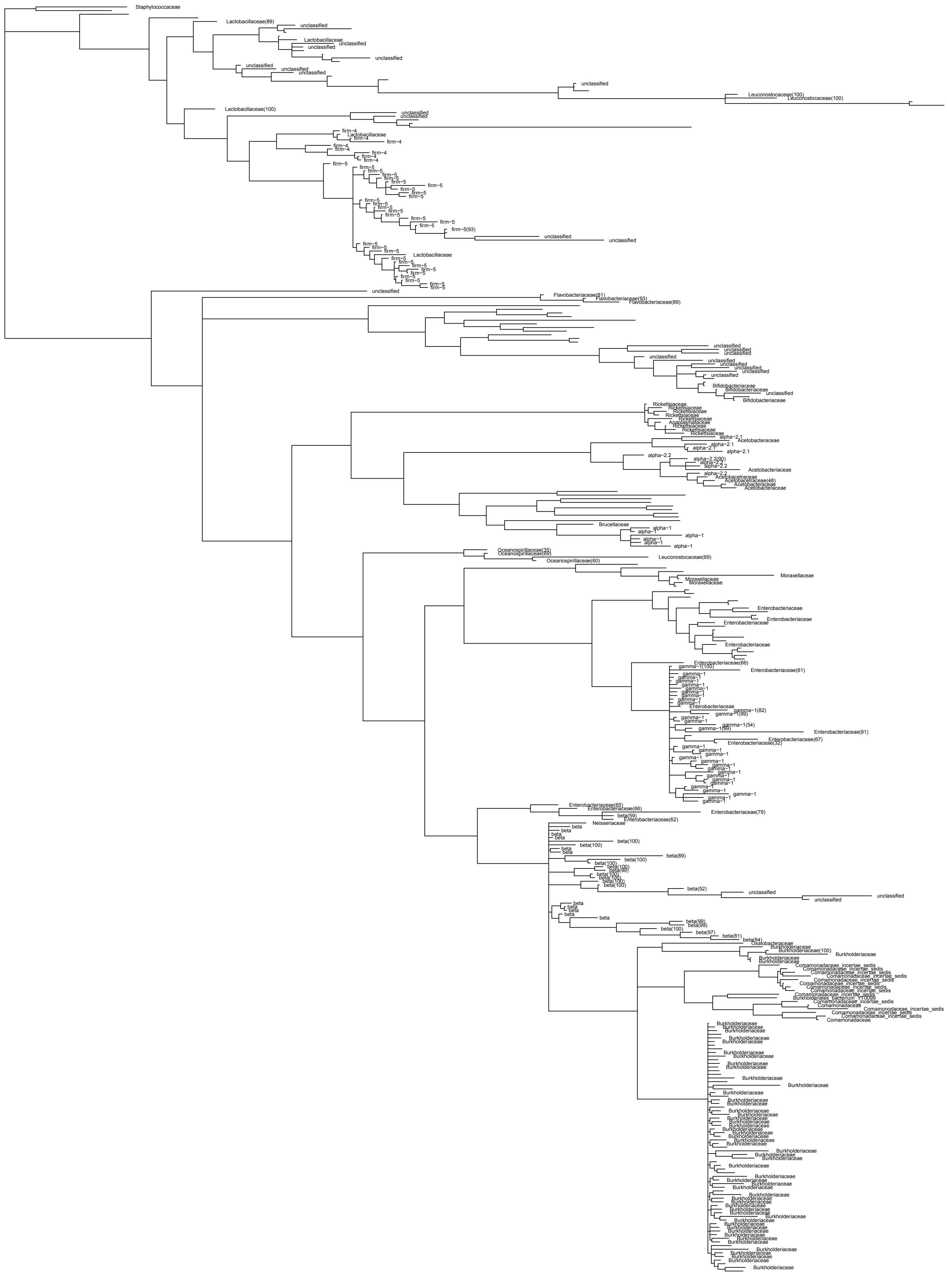
Staphylococcaceae
Lactobacillaceae(89)
unclassified
Lactobacillaceae
Leuconostocaceae(100)
Lactobacillaceae(100)
firm-4
firm-5
firm-5(93)
Flavobacteriaceae(81)
Flavobacteriaceae(93)
Flavobacteriaceae(89)
Bifidobacteriaceae
Rickettsiaceae
Anaplasmataceae
alpha-2.1
Acetobacteraceae
alpha-2.2
alpha-2.2(90)
Acetobacteraceae(48)
Brucellaceae
alpha-1
Oceanospirillaceae(35)
Oceanospirillaceae(69)
Oceanospirillaceae(60)
Leuconostocaceae(69)
Moraxellaceae
Enterobacteriaceae
Enterobacteriaceae(66)
gamma-1(100)
Enterobacteriaceae(61)
gamma-1
gamma-1(82)
gamma-1(99)
gamma-1(54)
Enterobacteriaceae(91)
Enterobacteriaceae(67)
Enterobacteriaceae(32)
Enterobacteriaceae(65)
Enterobacteriaceae(79)
beta(59)
Enterobacteriaceae(62)
Neisseriaceae
beta
beta(100)
beta(89)
beta(99)
beta(52)
beta(97)
beta(81)
beta(94)
Oxalobacteraceae
Burkholderiaceae
Burkholderiaceae(100)
Comamonadaceae_incertae_sedis
Comamonadaceae
Burkholderiales_bacterium
Burkholderiaceae
