# Supplementary material for: Deep Sequencing and Ecological Characterization of Gut Microbial Communities of Diverse Bumble Bee Species
Source: PLoS One. 2015 Mar 13;10(3):e0118566. doi: 10.1371/journal.pone.0118566 (PMC4359114; doi:10.1371/journal.pone.0118566)
Supplement: S1 File — (DOCX) [file pone.0118566.s006.docx]

**Supplemental Methods**

**Pyrosequencing library preparation**

Two rounds of PCR were conducted to produce sequencing libraries. For the first round of PCR (E515f and E1063r), each reaction (total volume = 10 ul) consisted of 2.3 mM MgCl_2_, 2 mM of each dNTP, 0.2 U Phusion Hi Fidelity Polymerase (NEB Biolabs), 2 μM of each primer and ~30 ng of template DNA. The thermocycling protocol used was: 10 cycles of 98 °C-1 min, 69 °C-1 min, 72 °C-45 s, followed by 25 cycles of 98 °C-30 s, 72 °C-45 s. For the second round of PCR, each reaction (reaction volume = 50 ul) consisted of 1.8 mM MgCl_2_, 2 mM of each dNTP, 1 U Phusion Hi Fidelity Polymerase and 2 μM of each the following primers. The forward primer contained (from 5’ to 3’) Roche 454 Lib-L fusion primer A, a 10-bp barcode and a 20-bp oligonucleotide that targets the universal tail adjacent to the E515f primer. The reverse primer contained (from 5’ to 3’) the Roche 454 Lib-L fusion primer B and a 20-bp portion that targets the other universal tail. The thermocycling protocol used was 10 cycles of 98 °C-1 min, 60 °C-1 min, 72 °C-45 s, followed by 15 cycles of 98 °C-30 s, 72 °C-45 s.

**Pyrosequencing data processing**

Initial quality filtering of the sequencing data (142,658 high quality sequences; average length = 609.9 bp), including flowgram-based denoising [1], reduced the number of sequences to 130,598 (number of unique sequences = 8,396, average length = 477.4 bp). The improved sequences were aligned to the custom, taxonomically annotated 16S rRNA training set using mothur’s align.seq, which relies on kmer searching, to carry out the alignment. After alignment, we used a pseudo-single linkage algorithm (mothur’s pre.cluster) to further reduce errors likely caused by pyrosequencing. Next, we conducted chimeric DNA detection and weeding using the program UCHIME implemented in mothur’s chimera.uchime [2]. Taxonomic classification of the remaining sequences (total number = 114,600, number of unique sequences = 1,732) was conducted with mothur’s classify.seq [3]. This method used taxonomic information contained within the entire training set and kmer searching to assign the most probable taxonomy to any given sequence. We used mothur’s remove.lineages to remove sequences with taxonomic designations matching cyanobacteria or chloroplast, and mothur’s split.abund.to remove sequences that were represented by just one read. We then took the remaining sequences (*n =* 106,196, average length = 445.0) and carried out average neighbor clustering to assign sequences to clusters (distance threshold = 3%).

**16S rRNA rarefaction analysis**

For intra-sample rarefaction analyses, we used a randomization procedure to resample without replacement (number of iterations = 1,000) sequences from a sequencing sample, and displayed the number of OTU’s represented as a function of the number of reads. For inter-sample rarefaction, we used the same re-sampling scheme (except that the number of resampling iterations was 100) to assess the effect of adding sequencing samples on the overall number of bacterial OTU’s discovered. For the intra-sample rarefaction analysis, we saved the rarefaction results for every 100 sequences.

**Sample preparation for microscopy of bumble bee gut tissues**

For light microscopy, bees were first anesthetized and submerged in Grace's insect culture medium. They were then dissected in Petri dishes with black Sylgard bottoms. Following dissection, the bees were fixed at 4 ºC in a primary fixative of 2.5 % glutaraldehyde and 0.5 % paraformaldehyde dissolved in a rinse buffer of 0.1 M cacodylate (pH 7.4) containing 0.18 mM CaCl_2_ and 0.58 mM sucrose. After three hours in primary fixative, tissues were washed three times with rinse buffer before being transferred to a secondary fixative (2% osmium tetroxide in rinse buffer). Tissues remained in this solution for four hours in the cold and were then washed three more times with rinse buffer. To enhance membrane contrast, rinsed tissues were placed in filtered, saturated uranyl acetate for 15 min immediately before being gradually dehydrated in a graded ethanol series (10%-100%). From absolute ethanol, tissues for sectioning were transferred to propylene oxide and infiltrated with mixtures of propylene oxide and resin before being embedded in pure LX112 resin. Resin was polymerized at 40ºC for one day and at 60ºC for three days. Embedded tissues were sectioned with a diamond knife at 1.0 µm and then floated onto glass slides covered with distilled water. After the water evaporated, the adherent sections were stained with a solution of 0.5% toluidine blue and 0.25% basic fuchsin in 1% borax.

To conduct fluorescent labeling of the outer cell walls of Gram-negative bacteria, whole mount of the gut was fixed in 4% paraformaldehyde (w/v) dissolved in phosphate-buffered saline (PBS, pH 7.4). After several rinses with PBS to remove residual fixative, tissues were permeabilized for at least 30 min by the addition of blocking buffer (PBS with 10% normal rabbit serum and 0.1% Triton X-100). The cells were subsequently labeled with addition of a 1:100 dilution of a primary goat antibody (Abnova) that recognizes the lipopolysaccharide (LPS) of Gram-negative bacteria. After an overnight incubation with primary antibody at 4ºC, tissues were rinsed at least three times with blocking buffer and then incubated for 2 h at room temperature with 7.5 mg/ml fluorescein isothiocyanate (FITC) coupled rabbit anti-goat antibody (Vector). Following three more rinses with blocking buffer, labeled tissues were mounted in 70% glycerin (v/v) in 0.1 M Tris (pH 9.0) [4].

**References**

1. Quince C, Lanzen A, Davenport R, Turnbaugh P (2011) Removing noise from pyrosequenced amplicons. BMC Bioinformatics 12: 38.

2. Edgar RC, Hass BJ, Clemente JC, Quince C, Knight R (2011) UCHIME improves sensitivity and speed of chimera detection. Bioinformatics 27: 2194-2200.

3. Wang Q, Garrity GM, Tiedje JM, Cole JR (2007) Naive Bayesian classifier for rapid assignment of rRNA sequences into the new bacterial taxonomy. Appl Environ Microbiol 73: 5261-5267.

4. Newton I, Roeselers G (2012) The effect of training set on the classification of honey bee gut microbiota using the Naive Bayesian Classifier. BMC Microbiol 12: 221.
