# Supplementary material for: Deep Sequencing and Ecological Characterization of Gut Microbial Communities of Diverse Bumble Bee Species
Source: PLoS One. 2015 Mar 13;10(3):e0118566. doi: 10.1371/journal.pone.0118566 (PMC4359114; doi:10.1371/journal.pone.0118566)
Supplement: S1 Table — Also provided are number of sequences that belong to each OTU. Numbers in parenthesis next to each taxonmic classification represents bootstrap confidence. (PDF) [file pone.0118566.s007.pdf]

S1 Table. Taxonomic classification of representative sequences of 74 OTU's obtained from 16S rRNA amplicon sequencing of 13 bumble bee whole guts. Also provided are number of sequences that belong to each OTU. Numbers in parenthesis next to each taxonmic classification represents bootstrap confidence.

Representative sequence	Class	Order	Family	No. of samples with the OTU	Total no. of reads	terricola_MI	terricola_WI	pensylvanicus_FL	pensylvanicus_NC	bohemicus	atratus	ussuriensis	convexus	fracternus_FL	impatiens_GA	impatiens_MI	terrestris_ARG	terrestris_FR
HUSOE0I01A3N3V	Betaproteobacteria(100)	Neisseriales(100)	beta(100)	12	57913	2184	26	11909	4775	320	8887	1	0	659	11242	2957	2821	12132
HUSOE0I01BDDRB	Bacilli(100)	Lactobacillales_incertae_sedis(100)	unclassified	5	14907	0	0	25	0	0	125	12152	0	0	0	1	2604	0
HUSOE0I01BWQDP	Gammaproteobacteria(100)	Enterobacteriales(100)	gamma-1(100)	9	11601	1102	8320	11	16	0	143	0	0	1931	1	0	7	70
HUSOE0I01BNGHK	Flavobacteria(93)	Flavobacteriales(93)	Flavobacteriaceae(93)	7	10853	1273	850	1	1	0	0	0	56	8602	0	0	0	70
HUSOE0I01BUA5R	Bacilli(100)	Lactobacillales(100)	Leuconostocaceae(100)	4	3552	0	0	0	15	0	0	3	3511	0	0	0	23	0
HUSOE0I01BOC8I	Bacilli(100)	Lactobacillales_incertae_sedis(95)	unclassified	2	2665	0	0	0	46	0	0	0	2619	0	0	0	0	0
HUSOE0I01AJO2K	Actinobacteria(100)	Bifidobacteriales(88)	unclassified	7	1288	13	1	56	123	0	264	0	0	49	0	782	0	0
HUSOE0I01BVLCI	Gammaproteobacteria(100)	Oceanospirillales(70)	Oceanospirillaceae(69)	1	783	0	0	0	0	0	0	0	783	0	0	0	0	0
HUSOE0I01AYGNK	Alphaproteobacteria(100)	Rhodospirillales(90)	Acetobacetraceae(48)	2	641	0	0	0	0	640	0	0	0	0	0	0	1	0
HUSOE0I01A8PYF	Gammaproteobacteria(67)	Enterobacteriales(67)	Enterobacteriaceae(32)	3	612	0	0	0	0	321	0	0	0	0	119	172	0	0
HUSOE0I01A3MOH	Actinobacteria(100)	Bifidobacteriales_incertae_sedis(99)	unclassified	3	567	0	0	0	0	0	0	0	0	0	5	505	0	57
HUSOE0I01B3JGQ	Betaproteobacteria(80)	Neisseriales(80)	beta(59)	3	195	0	0	0	0	4	0	0	0	0	120	71	0	0
HUSOE0I01BFNX7	Betaproteobacteria(100)	Neisseriales(100)	beta(100)	6	143	17	0	2	0	0	0	0	0	0	60	14	1	49
HUSOE0I01BP7S6	Bacilli(93)	Lactobacillales(93)	firm-5(93)	9	92	16	12	0	5	2	0	17	0	8	0	26	4	2
HUSOE0I01B32PS	Betaproteobacteria(100)	Burkholderiales(100)	Burkholderiaceae(100)	3	42	0	0	1	0	7	0	34	0	0	0	0	0	0
HUSOE0I01B2I67	Betaproteobacteria(100)	Neisseriales(100)	beta(100)	3	42	0	0	36	2	0	4	0	0	0	0	0	0	0
HUSOE0I01BUQ2B	Betaproteobacteria(100)	Neisseriales(100)	beta(100)	4	31	0	0	16	1	0	9	0	0	5	0	0	0	0
HUSOE0I01CE7GA	Betaproteobacteria(98)	Neisseriales(98)	beta(97)	3	22	0	0	7	4	0	0	0	0	0	0	0	0	11
HUSOE0I01A3DA4	Gammaproteobacteria(85)	Enterobacteriales(85)	Enterobacteriaceae(65)	3	22	0	5	0	0	0	0	0	0	15	0	0	0	2
HUSOE0I01BWY8W	Bacilli(100)	Lactobacillales_incertae_sedis(100)	unclassified	1	18	0	0	0	0	0	0	0	0	0	0	0	18	0
HUSOE0I01BRGR9	Bacilli(100)	Lactobacillales_incertae_sedis(99)	unclassified	1	14	0	0	0	0	0	0	0	0	0	0	0	14	0
HUSOE0I01BWUY5	Betaproteobacteria(100)	Neisseriales(100)	beta(99)	4	13	1	0	0	0	0	0	0	0	0	3	4	0	5
HUSOE0I01ATT37	Gammaproteobacteria(100)	Enterobacteriales(100)	gamma-1(82)	3	13	7	3	0	0	0	0	0	0	3	0	0	0	0
HUSOE0I01BZVWB	Betaproteobacteria(100)	Neisseriales(100)	beta(100)	3	9	0	0	2	6	0	1	0	0	0	0	0	0	0
HUSOE0I01A9P4E	Bacilli(99)	Lactobacillales(89)	Lactobacillaceae(89)	1	8	0	0	0	0	0	0	8	0	0	0	0	0	0
HUSOE0I01CDEN5	Betaproteobacteria(95)	Neisseriales(92)	beta(81)	2	7	0	0	2	0	0	0	0	0	0	0	0	5	0
HUSOE0I01CD6EU	Betaproteobacteria(99)	Neisseriales(99)	beta(99)	2	7	0	0	0	0	0	0	0	0	0	4	0	0	3
HUSOE0I01BP6WJ	Bacilli(100)	Lactobacillales_incertae_sedis(94)	unclassified	1	7	0	0	0	0	0	0	0	0	0	0	0	7	0
HUSOE0I01BD200	Actinobacteria(100)	Bifidobacteriales(88)	unclassified	1	7	0	0	0	0	0	0	7	0	0	0	0	0	0
HUSOE0I01AWCV6	Bacilli(100)	Lactobacillales_incertae_sedis(93)	unclassified	1	7	0	0	0	0	0	0	0	0	0	0	0	7	0
HUSOE0I01BCP6C	Actinobacteria(100)	Bifidobacteriales_incertae_sedis(99)	unclassified	1	6	0	0	0	0	0	0	0	0	0	0	6	0	0
HUSOE0I01BBJAD	Bacilli(100)	Lactobacillales(100)	Leuconostocaceae(100)	1	5	0	0	0	0	0	0	0	5	0	0	0	0	0
HUSOE0I01AZRX1	Betaproteobacteria(100)	Neisseriales(100)	beta(100)	1	5	0	0	0	5	0	0	0	0	0	0	0	0	0
HUSOE0I01CBAJ8	Gammaproteobacteria(100)	Enterobacteriales(100)	gamma-1(54)	1	4	0	4	0	0	0	0	0	0	0	0	0	0	0
HUSOE0I01BW3DE	Betaproteobacteria(99)	Neisseriales(99)	beta(99)	2	4	0	0	3	0	0	1	0	0	0	0	0	0	0
HUSOE0I01AHDHH	Gammaproteobacteria(99)	Oceanospirillales(62)	Oceanospirillaceae(60)	1	4	0	0	0	0	0	0	0	4	0	0	0	0	0
HUSOE0I01A4VJH	Betaproteobacteria(97)	Neisseriales(95)	beta(89)	1	4	0	0	0	0	0	0	0	0	4	0	0	0	0
HUSOE0I01CDMUK	Bacilli(100)	Lactobacillales(100)	Lactobacillaceae(100)	1	3	0	0	0	0	0	0	3	0	0	0	0	0	0
HUSOE0I01BJ01Q	Gammaproteobacteria(80)	Enterobacteriales(79)	Enterobacteriaceae(79)	1	3	0	0	0	0	0	0	0	0	3	0	0	0	0
HUSOE0I01BB9C3	Bacilli(62)	Lactobacillales_incertae_sedis(61)	unclassified	1	3	0	0	0	0	0	0	0	0	0	0	0	0	3
HUSOE0I01B240B	ML635J-21(21)	unclassified	unclassified	1	3	0	0	0	0	0	0	3	0	0	0	0	0	0
HUSOE0I01B0TB0	Gammaproteobacteria(75)	Enterobacteriales(75)	Enterobacteriaceae(66)	1	3	0	0	3	0	0	0	0	0	0	0	0	0	0
HUSOE0I01ARNEE	Gammaproteobacteria(98)	Enterobacteriales(97)	Enterobacteriaceae(91)	1	3	0	0	0	0	0	0	0	0	3	0	0	0	0
HUSOE0I01AQ15O	Alphaproteobacteria(100)	Rhodospirillales(90)	alpha-2.2(90)	1	3	0	0	0	0	0	0	3	0	0	0	0	0	0
HUSOE0I01A7U5H	Betaproteobacteria(100)	Neisseriales(100)	beta(100)	1	3	0	0	0	0	0	0	0	0	0	0	0	0	3
HUSOE0I01A72EZ	Actinobacteria(59)	Bifidobacteriales_incertae_sedis(53)	unclassified	1	3	0	0	0	0	0	0	0	0	0	0	3	0	0
HUSOE0I01CD3QW	Gammaproteobacteria(100)	Enterobacteriales(100)	gamma-1(99)	1	2	0	2	0	0	0	0	0	0	0	0	0	0	0
HUSOE0I01CCSFS	Bacilli_incertae_sedis(41)	unclassified	unclassified	1	2	0	0	0	2	0	0	0	0	0	0	0	0	0
HUSOE0I01CBNPP	Actinobacteria(100)	Bifidobacteriales_incertae_sedis(82)	unclassified	1	2	0	0	0	0	0	0	0	0	0	0	2	0	0
HUSOE0I01BZKLU	Flavobacteria(82)	Flavobacteriales(82)	Flavobacteriaceae(81)	1	2	0	0	0	0	0	0	0	0	2	0	0	0	0
HUSOE0I01BWBQH	Bacilli(88)	Lactobacillales_incertae_sedis(53)	unclassified	1	2	0	0	2	0	0	0	0	0	0	0	0	0	0
HUSOE0I01BRHWZ	Actinobacteria(96)	Bifidobacteriales(71)	unclassified	1	2	0	0	2	0	0	0	0	0	0	0	0	0	0
HUSOE0I01BKM8G	Actinobacteria(100)	Bifidobacteriales(66)	unclassified	1	2	0	0	0	0	0	0	0	0	0	0	2	0	0
HUSOE0I01BJ87N	Betaproteobacteria(100)	Neisseriales(100)	beta(100)	1	2	0	0	0	0	0	0	0	0	0	0	2	0	0
HUSOE0I01BC4E4	Betaproteobacteria(100)	Neisseriales(100)	beta(100)	1	2	0	0	0	2	0	0	0	0	0	0	0	0	0
HUSOE0I01B8SEI	Bacilli(100)	Lactobacillales_incertae_sedis(100)	unclassified	1	2	0	0	0	0	0	0	0	0	0	0	0	2	0
HUSOE0I01B7PJE	Betaproteobacteria(99)	Neisseriales(99)	beta(94)	1	2	0	0	0	0	0	0	0	0	0	0	0	2	0
HUSOE0I01B5IQC	Gammaproteobacteria(67)	Enterobacteriales(67)	Enterobacteriaceae(66)	1	2	0	0	0	0	0	0	0	0	0	2	0	0	0
HUSOE0I01AXW23	Bacilli(84)	Lactobacillales_incertae_sedis(60)	unclassified	1	2	0	0	2	0	0	0	0	0	0	0	0	0	0
HUSOE0I01AWST0	Bacilli(100)	Lactobacillales_incertae_sedis(100)	unclassified	1	2	0	0	0	2	0	0	0	0	0	0	0	0	0
HUSOE0I01AW46E	Actinobacteria(100)	Bifidobacteriales_incertae_sedis(97)	unclassified	1	2	0	0	0	0	0	0	0	0	0	0	2	0	0
HUSOE0I01AVA9Y	Actinobacteria(100)	Bifidobacteriales_incertae_sedis(69)	unclassified	1	2	0	0	0	0	0	0	0	0	0	0	2	0	0
HUSOE0I01AV2VD	Gammaproteobacteria(97)	Enterobacteriales(97)	Enterobacteriaceae(61)	1	2	0	2	0	0	0	0	0	0	0	0	0	0	0
HUSOE0I01ATNJR	Gammaproteobacteria(97)	Oceanospirillales(39)	Oceanospirillaceae(35)	1	2	0	0	0	0	0	0	0	2	0	0	0	0	0
HUSOE0I01ASHHP	Bacilli(100)	Lactobacillales_incertae_sedis(100)	unclassified	1	2	0	0	2	0	0	0	0	0	0	0	0	0	0
HUSOE0I01ANFEB	Bacilli(74)	Lactobacillales(72)	Leuconostocaceae(69)	1	2	0	0	0	0	0	0	0	2	0	0	0	0	0
HUSOE0I01AMOCR	Actinobacteria(100)	Bifidobacteriales(87)	unclassified	1	2	0	0	0	0	0	0	2	0	0	0	0	0	0
HUSOE0I01AMI8L	Gammaproteobacteria(67)	Enterobacteriales(67)	Enterobacteriaceae(67)	1	2	0	0	0	0	2	0	0	0	0	0	0	0	0
HUSOE0I01AIU2N	Gammaproteobacteria(100)	Enterobacteriales(100)	gamma-1(99)	1	2	0	2	0	0	0	0	0	0	0	0	0	0	0
HUSOE0I01AIB7F	Flavobacteria(89)	Flavobacteriales(89)	Flavobacteriaceae(89)	2	2	1	0	0	0	0	0	0	0	0	0	0	0	1
HUSOE0I01A7CXH	Betaproteobacteria(88)	Neisseriales(88)	beta(52)	1	2	0	0	2	0	0	0	0	0	0	0	0	0	0
HUSOE0I01A5OX9	Betaproteobacteria(100)	Neisseriales(100)	beta(100)	1	2	0	0	0	0	0	0	0	0	0	0	0	2	0
HUSOE0I01A5EKU	Gammaproteobacteria(69)	Enterobacteriales(69)	Enterobacteriaceae(62)	1	2	2	0	0	0	0	0	0	0	0	0	0	0	0
HUSOE0I01A3VJ4	Bacilli(100)	Lactobacillales_incertae_sedis(100)	unclassified	1	2	0	0	0	0	0	0	0	0	0	0	0	2	0
				Sum	106196	4616	9227	12084	5005	1296	9434	12233	6982	11284	11556	4551	5520	12408
